# Meerkat close calling patterns are linked to sex, social category, season and wind, but not fecal glucocorticoid metabolite concentrations

**DOI:** 10.1371/journal.pone.0175371

**Published:** 2017-05-03

**Authors:** Jelena Mausbach, Ines Braga Goncalves, Michael Heistermann, André Ganswindt, Marta B. Manser

**Affiliations:** 1 Animal Behaviour, Department of Evolutionary Biology and Environmental Studies, University of Zurich, Zurich, Switzerland; 2 Kalahari Meerkat Project, Kuruman River Reserve, Van Zylsrus, Northern Cape, South Africa; 3 Endocrinology Laboratory German Primate Center, Göttingen, Germany; 4 Endocrine Research Laboratory, Dept. of Anatomy & Physiology, Faculty of Veterinary Science, University of Pretoria, Onderstepoort, South Africa; 5 Mammal Research Institute, University of Pretoria, Hatfield, South Africa; Tierarztliche Hochschule Hannover, GERMANY

## Abstract

It is well established that animal vocalizations can encode information regarding a sender’s identity, sex, age, body size, social rank and group membership. However, the association between physiological parameters, particularly stress hormone levels, and vocal behavior is still not well understood. The cooperatively breeding African meerkats *(Suricata suricatta)* live in family groups with despotic social hierarchies. During foraging, individuals emit close calls that help maintain group cohesion. These contact calls are acoustically distinctive and variable in rate across individuals, yet, information on which factors influence close calling behavior is missing. The aim of this study was to identify proximate factors that influence variation in call rate and acoustic structure of meerkat close calls. Specifically, we investigated whether close calling behavior is associated with sex, age and rank, or stress hormone output (i.e., measured as fecal glucocorticoid metabolite (fGCM) concentrations) as individual traits of the caller, as well as with environmental conditions (weather) and reproductive seasonality. To disentangle the effects of these factors on vocal behavior, we analyzed sound recordings and assessed fGCM concentrations in 64 wild but habituated meerkats from 9 groups during the reproductive and non-reproductive seasons. Dominant females and one-year old males called at significantly higher rates compared to other social categories during the reproductive season. Additionally, dominant females produced close calls with the lowest mean fundamental frequencies (F0) and the longest mean pulse durations. Windy conditions were associated with significantly higher call rates during the non-reproductive season. Fecal GCM concentrations were unrelated to close calling behavior. Our findings suggest that meerkat close calling behavior conveys information regarding the sex and social category of the caller, but shows no association with fGCM concentrations. The change in call rate in response to variation in the social and ecological environments individuals experience indicates some degree of flexibility in vocal production.

## Introduction

Vocal communication mediates many interactions between individuals in social species. Therefore, identifying factors that might cause variation between and within individuals in vocal production can help us understand species’ social dynamics. Call types are typically context-specific, yet often vary in call rate and acoustic structure between and within individuals, due to genetic, anatomical and/or physiological differences [1–6]. Vocalizations can convey information concerning an individual’s identity, sex, age, social status, group membership, body size, and physiological condition [1, 7, 8]. While acoustic properties that determine individual distinctiveness are genetically determined, other aspects of calls, such as those caused by physiological parameters [8], can be more or less flexible [9, 10]. Individual physiological [e.g. hormonal) states related to sex, age or social status [11–14] can be stable over long periods of time, but can also change within short periods of time in response to e.g. reproductive state [11], social instability or environmental challenges, such as predation risk, food limitation and harsh weather conditions [15–18].

Previous research on hormonal mediation of vocal communication has established a modulatory role of reproductive hormones on vocal production rate and acoustic structure in amphibians, birds, and mammals [19–25]. In contrast, the influence of hormones involved in the stress response on vocal behavior and acoustic structure, has remained largely unexplored. Such acoustic cues linked to physiological indicators of stress may enable conspecifics to monitor other individuals’ physiological and motivational states, and play a role in the mediation of conflicts or management of relationships. Glucocorticoids (GCs), the class of steroid hormones that includes cortisol and corticosterone, are closely associated with an organism’s response to internal and external stressors, and likely affect vocal production via GC receptors in the vocal tract [7]. For example, in rock hyraxes (*Procavia capensis*), males that have higher reproductive success also have higher corticosterone levels and sing more [26]. Several studies have shown that high arousal and stressful states, which are assumed to increase GC concentrations, can affect vocal production in vertebrates [3, 4, 26–29]. For instance, fundamental frequency (F0), the lowest frequency of a periodic waveform, tends to be higher in calls produced in high-arousal contexts, such as during encounters with predators [2, 30]. However, these studies have not monitored concentrations of GCs or their excreted metabolites, and it is unclear whether there is a direct link between GC output and vocal production, or whether GCs association to acoustic changes is mediated by other components of an individual’s physiological stress response [30]. Elevated plasma cortisol levels, e.g. induced by prolonged physically demanding situations such as reproduction, lactation or starvation [11, 31] can be detected through corresponding elevations in fecal GC metabolites (fGCMs) [32, 33]. The temporal delay in the appearance of the respective signal in the feces as well as the cumulative secretion of fGCMs allows researchers to determine more accurately individual levels of stress hormone output that may be indicative of perceived chronic stressors [34–37].

Meerkats are cooperatively breeding carnivores that live in despotic groups of 2 to 50 individuals, consisting of a dominant pair and subordinates [38]. The dominant pair monopolizes reproduction within the group leading to high intra-sexual conflict. This is particularly true for females, as dominant females commonly evict the eldest subordinate and other females during the late stages of pregnancy [39–41]. Dominant females also behave more aggressively in food competition contexts during reproductive periods, presumably as a result of their higher energetic needs [39, 40]. Mean fGCM levels (with a temporal delay of about 24 hrs in meerkats reflecting plasma cortisol concentrations [33]) are associated with higher reproductive rates [11] and with vigilance behavior [30]. Meerkats reach sexual maturity around one year of age, although they continue to grow for a while longer [42]. Males are the dispersing sex and usually leave their natal groups around the age of 2 years [41, 43]. However, in groups where subordinate males are not related to the dominant female, substantial intra-sexual competition can also occur between the dominant and subordinate males [44]. In both sexes, conflict increases with subordinate age [45]. Meerkats spend a large proportion of their time foraging for food as a cohesive unit, with heads down digging for prey in the sand [46]. All group members produce low amplitude contact calls at short distances from neighbors, termed ‘close calls’ [6, 47], which help maintain group cohesion [48, 49]. Individuals show high variation in calling rate [49] and in the acoustic structure of their calls [6], and they can discriminate between group members based on acoustic cues of close calls [5]. For example, dominant females emit distinctive close calls that cause other group members to adjust their own behavior [50]. The distinctiveness of dominant females’ close calls may be related to their typically larger body sizes, which in other species have been associated with longer, thicker and looser vocal folds resulting in lower fundamental frequencies [51–53]. Moreover, at 3–5 months of age, juveniles’ begging calls change into close calls given at much higher rates than those of adults [54].

To improve our understanding of the observed within- and between-individual variation in close calling behavior, we investigated several proximate factors that may affect close call production in wild meerkats during foraging through behavioral observations and acoustic analyses. By focusing on close calls produced in the foraging, context rather than on alarm calls produced under perceived predator threat, we aimed to minimize potential confounding effects from other components of an animal’s physiological stress response, allowing us to evaluate whether call rate and acoustic structure were associated with individual traits (sex, age and social rank), weather conditions, reproductive seasonality, and fGCM levels. Overall, we aimed to answer the following questions: Are individual traits such as sex, age, social rank or fGCM levels, as well as weather conditions (cloud cover and wind) correlated with i) close call rate, and ii) the acoustic structure of close calls? Further, iii) are the above relationships affected by seasonal context (reproductive vs non-reproductive), and iv) are fGCM levels influenced by traits such as sex, age and social rank?

We predicted that close call rate is influenced by sex (due to sex-differences in the amount of GC receptors, [55]) and social category, as males and females as well as dominants and subordinates are exposed to distinct social challenges. We also predicted calls of the dominant and larger individuals to have lower fundamental frequencies [52, 53, 56–59]. We expected close call rate and mean fundamental frequency of calls to be positively correlated with fGCM concentrations [2], in general, as well as specifically during unfavorable weather conditions. Overcast skies decrease predator visibility and windy conditions reduce the detectability of conspecific calls, and therefore both conditions were expected to be stressful for meerkats. Finally, we predicted that close call rate, structure and fGCM levels would differ between social categories and also between reproductive and non-reproductive seasons because of differences in the social (e.g. intra-sexual competition) and intrinsic (e.g. hormonal changes due to reproductive state) challenges individuals experience.

## Material and methods

### Study site and population

The study was conducted at the Kalahari Meerkat Project (KMP) in South Africa (26°58’S, 21°49’E) [52]. All animals in the population were known individually, as well as their group affiliations, age, social rank and life history except for a few immigrants (< 5% of the population). Animals were tagged with transponder microchips (Identipet^®^, Johannesburg, South Africa) for lifetime individual recognition and marked with unique hair dye patterns for immediate visual identification [53]. Groups were habituated to human presence to a level that they could be followed and recorded from as close as one meter. The study was conducted within a general permission of the ethical committee for animal research, from the University of Pretoria (EC011-10) and the Northern Cape Conservation Service, South Africa.

### Study design and data collection

We sampled nine meerkat groups with group sizes between 7 and 37 individuals. Originally we aimed to measure close call rate, call structure and fGCM concentrations in eight meerkats per group. However, as one group only consisted of seven individuals and other individuals died or left the group during the study, the total number of animals sampled for the reproductive and non- reproductive season resulted in N = 66 and N = 64, respectively (exact N for each variable measured is given in the respective Figs and Table legends). Focal subjects were classified depending on sex and social rank, and within subordinates also by age into the following social categories [60]: the dominant pair, the eldest subordinate, and two-year olds and one-year olds subordinate of each sex. Among subordinates, focal individuals of the same social category were littermates whenever possible. We aimed for balanced sampling across social categories in all groups (S3 Table).

Data were collected during two seasons that differed with regards to the reproductive state of the females and the presence of dependent offspring in the groups. The first part of the study was conducted between February and March 2012, during peak reproductive season, when the majority of the litters were born and most dominant females were lactating. The litters were approximately one to two weeks old on the first sampling day and were not foraging with the groups during the period of data collection. The second part of the study was conducted between April and July 2012 during the non-reproductive season, when the youngest individuals (juveniles, 3 to 6 months) in the groups were at least 3 months old and only seldom begging for food while foraging [54]. Females were neither lactating nor obviously pregnant. The exact dates of data collection for sound, behavior and fecal samples can be found in the S4 Table.

#### Behavioral observations

Observations took place in the mornings once the meerkats started foraging after leaving the sleeping burrow. In each season, data were collected on two separate sessions per group, approximately one week apart. On the first morning, focal individuals were observed in random order; on the second morning, the order was reversed to avoid order effects related to changes in foraging behavior over the morning. If a focal individual was not foraging with the group on a focal day, the group was visited again as soon as possible to collect data of the “missing” individual.

To assess close call rate, focal individuals were observed for 10 minutes during foraging, recording their behavior and each call produced on a data logger (PSION Organizer II Model LZ64, DPP Focal Protocol). Noted behaviors are described in the ethogram following ([49], S1 Table). To investigate close call structure, individuals were recorded for 5–10 minutes using a directional microphone (Sennheiser ME66 with K6 powering module) fixed to a tripod leg connected to a solid state recorder (Marantz PMD660, sampling frequency 44.1 kHz, 16 bit, frequency response: 20Hz - 20kHz), while on the second channel we used a second microphone (Joseph E-280 Dynamic Microphone, 40 Hz—20 kHz, (+/- 2.5 dB)) to continuously describe the focal individual’s behavior and the context in which each call was produced. In addition, in each session we measured wind speed with a wind meter (Kaindl-electronic Windmaster II) and categorized it as a binary variable “yes” (> 0.5 m/s) or “no” (< 0.5 m/s); and we classified weather as “good” if < 30% cloud cover or overcast if > 30% cloud cover. Furthermore, we recorded the focals’ morning weight (using Sartorius TE4100 ±1g scales [42]), the reproductive status of the focal females (lactating: yes/no), whether subordinates had access to unrelated breeding partners within their group (yes/no [61]) and whether there were begging juveniles within 5 meters of the focal individual. For each focal subject, mean body mass was calculated using at least three morning weight values recorded within a week of each sampling day. To keep social conditions similar between the focal individuals, observations were not conducted if the group had split into subgroups. In case of naturally occurring alarm calls, observations were interrupted until the group had resumed normal foraging behavior for at least 10 minutes. When a focal individual went on raised guard or bipedal guard (S1 Table) for longer than one minute, observations were paused until the individual resumed normal foraging behavior.

#### Feces collection

Freshly deposited feces were collected for fGCM analysis throughout the reproductive (January to March) and non-reproductive season (April to July) (see S4 Table). Samples were taken immediately after the focal animal had left the defecation spot. On average, 4.0 (range 1–5) samples per individual were collected, 2.2 and 1.8 samples in the reproductive and non-reproductive season, respectively. We could not collect fecal samples from all meerkats on the same days as acoustic data were collected. Meerkats do not defecate frequently, and especially dominant individuals often defecate in burrows, making it impossible to collect their samples in these situations. However, by measuring fGCM, we aimed to assess associations between vocal behavior and potential long-term stressors only. As such, we considered the collection of fecal samples within the same reproductive season as the sound recordings to appropriately reflect the effects of combined stressors on vocal behavior. Samples were stored immediately in plastic bags and transported on ice in a thermos flask to the field base where they were stored at -20°C [62].

### Analysis on vocal production

#### Call rate

To calculate close call rate (number of calls/minute) we counted the total number of calls produced in one sampling session (10 minutes/focal) during foraging, including the focal moving, searching for food, digging, eating, and processing food, and divided it by the observation time. Call rates were calculated for each single sampling session for each individual (individuals in reproductive season: *N*_repID_ = 66, individuals in non-reproductive season *N*_nonrepID_ = 64), resulting in two data points for each individual per season.

#### Call structure

Sound files were visually assessed for call quality using Cool Edit 2000 Version 1.1 (Syntrillum Software Corporation). Whenever possible, seven good quality close calls per individual and sampling session (little background noise, short distance of 0.3 to 1.5 m to microphone) from digging or foraging contexts from the first 5 minutes of the sound file were copied into individual sound files and visually reassessed for quality as spectrograms and wave forms using Praat v5.3.03 (Amsterdam, Netherland). All following steps for the acoustic analysis were carried out using Praat.

**Fig 1:**
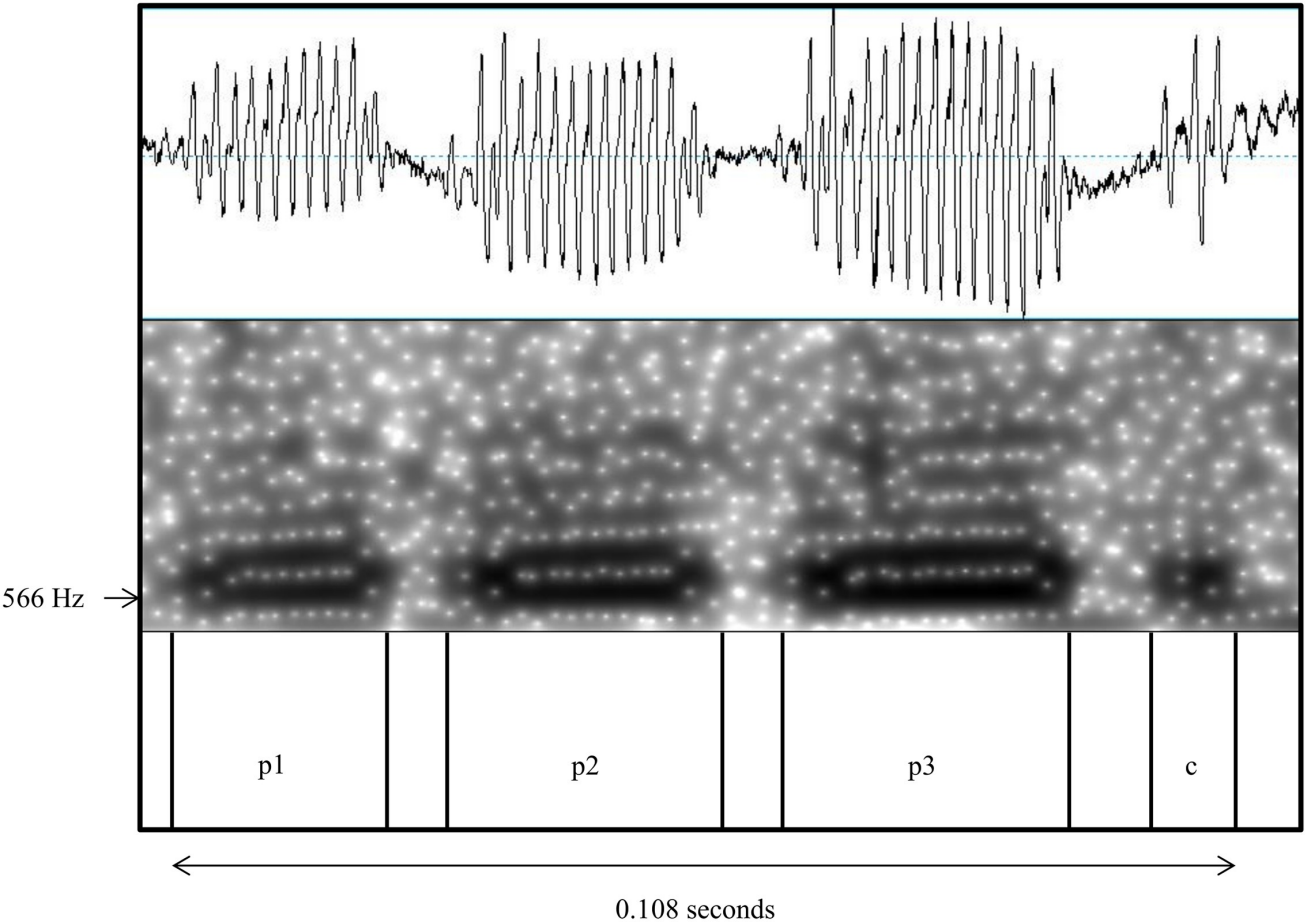
Waveform (top) and spectrogram (middle) of a close call. Example of a labelled close call with a “click” (c) (voiceless segment) at the end and 3 pulses (pi).

The selected calls were labelled using a text grid in order to make every step of the analysis reproducible. Every “pulse” of a call was labelled with “p*i*”, which is a noise sub-element, or with “c*i*” if it was a voiceless segment or ‘click’ (*i* = number of pulses or clicks) (Fig 1). Calls were saved as wave files with text grids. A filtering process was applied to sounds below 100 Hz to reduce background noise. A script (written by Volker Dellwo) was run using the following settings: pitch settings range: 300–1000 Hz, view range: 0–1000 Hz, frequency step of FFT 200 Hz. For each call the following parameters were measured: 1) Temporal parameters: number of call elements (pulses (ps) and clicks (cs))); call duration (seconds); mean pulse duration (seconds); mean inter-pulse interval (IPI) duration (seconds). 2) Spectrum-related parameters: mean (Hz) of the F0. F0 was controlled visually for its computed validity, before the script was run. Elements that were marked as F0, but were not part of the call or were shown as F1 or F2 instead of F0 were unvoiced and not considered later in the script. Mean values per day and individual were calculated for all acoustic call parameters for the statistical analyses.

### Endocrine analyses

#### Fecal steroid extraction

Steroid extractions were done at the Endocrine Research Laboratory, Faculty of Veterinary Science, University of Pretoria, SA, in November 2012, following previously described methods [63]. Following lyophilisation, samples were pulverized, and subsequently 0.10–0.11 g of fecal powder extracted with 3 ml 80% watery methanol by vortexing for 10 minutes on a multi-tube vortex [64]. Samples were then centrifuged for 10 minutes at 1500 *g*. Two 1 ml portions of the supernatant were stored in polypropylene microtubes, and one of these portions were oven dried [65, 66] for 14 hours at 55 to 58°C and shipped to the Endocrinology Laboratory of the German Primate Centre, Göttingen, Germany, for hormone assay.

#### 11ß-hydroxyetiocholanolone assay

All fecal extracts were measured for immunoreactive 11ß-hydroxyetiocholanolone, a major metabolite of cortisol in meerkats [33]. The measurement has recently been validated for monitoring glucocorticoid output in meerkats [33], and has also been successfully used to monitor adrenocortical activity in numerous other vertebrate species [66–72]. Prior to assay, dried fecal extracts were reconstituted in 0.5 ml 80% methanol by sonication in a water bath for 5 minutes, followed by 30 seconds of vortexing [66]. The assay was performed on microtiter plates following the procedure described in Heistermann et al. [68, 73]. Sensitivity of the assay was 1.2 pg. Cross-reactivities of the antibody used and further details of the EIA are reported in [67]. Intra-assay coefficients of variation (CV) for low- and high-value quality controls were 7.0% and 5.3%, respectively. Respective inter-assay CV values were 12.0% and 10.7%. All fGCM concentrations reported are expressed as ng/g dry fecal mass and as median of an individual for each season.

### Statistics

All statistics were done in R Studio Version 0.96.311 (R version 3.0.1.). Close call rate, all structural variables of the calls and fGCM concentrations were log-transformed to conform to parametric assumptions. Normality was tested by graphic analysis of the distribution of residuals. The structural variables were tested for collinearity (Pearson) and number of elements was excluded from further analyses because it was significantly positively correlated to total call duration (Pearson, cor = 0.85, *P* < 0.001). Linear mixed models (marginal p-values, Maximum Likelihood (ML), package in R: nlme, model type in R: lme) were used to assess the effects of the following fixed factors (FF): sex, social category (a composite measure of social rank and age), season and all possible interactions between these three factors, and additionally habitat, weather, wind and lactation state, on call rate, on each call structure variable and on fGCM concentrations. In all analyses of vocal variables, including rate and structure, mean body weight and median fGCM concentrations were included as covariates. Individual and group were included in all models as random factors (RF) to account for repeated sampling. Additionally, sampling term (RF) was included in the call rate and all call structure models, to correct for the fact that calling rate and structure may be more similar on sampling dates that were closer to each other. Significance of factors was tested using stepwise backward selection until the model only contained factors that would matter for the question of the study (social category, sex, season). Thus, the full model was progressively reduced by removing factors and interactions, one at a time, in case of non-significant ANOVA (marginal) comparisons. Only the minimal models or the models presenting significant factors are presented in the results section. In the case of significant seasonal effects, subsequent separate models were run for each season. Preliminary exploration of the relationship between fGCM levels and the vocal parameters was done using Pearson correlations (S6 Table). To describe patterns of significance within factors, differences in slope were shown graphically and post-hoc Tukey tests were run and are presented in the supplementary material (S2 Table and S1–S4 Figs). Significance was set at α ≤ 0.05 and all tests were two-tailed.

## Results

### Close call rate

**Fig 2:**
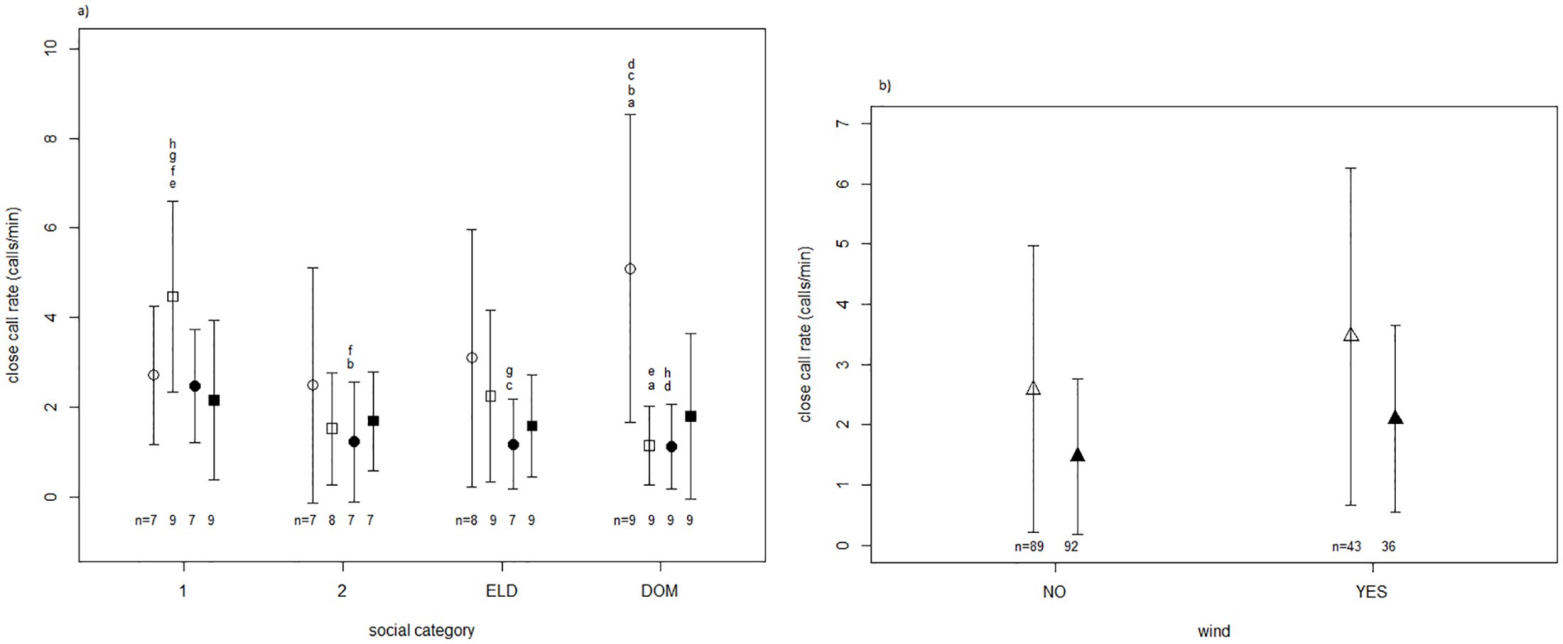
Mean close call rate (calls/minute). a) for each social category (1: one-year old subordinates, 2: two-year old subordinates, ELD: eldest subordinate present in group, DOM: dominant individuals), sex (female: ○, male □) and season (reproductive (white), non-reproductive (black)). N gives the number of individuals per condition. b) depending on wind for the reproductive (white) and non-reproductive (black) season. Whiskers show the standard deviation of the data. N gives the number of individuals (including replicate) sampled per condition. Graphs show untransformed data although the statistical tests were conducted on transformed data and as part of a mixed-model. Same letters indicate significant differences in posthoc tests.

**Fig 3:**
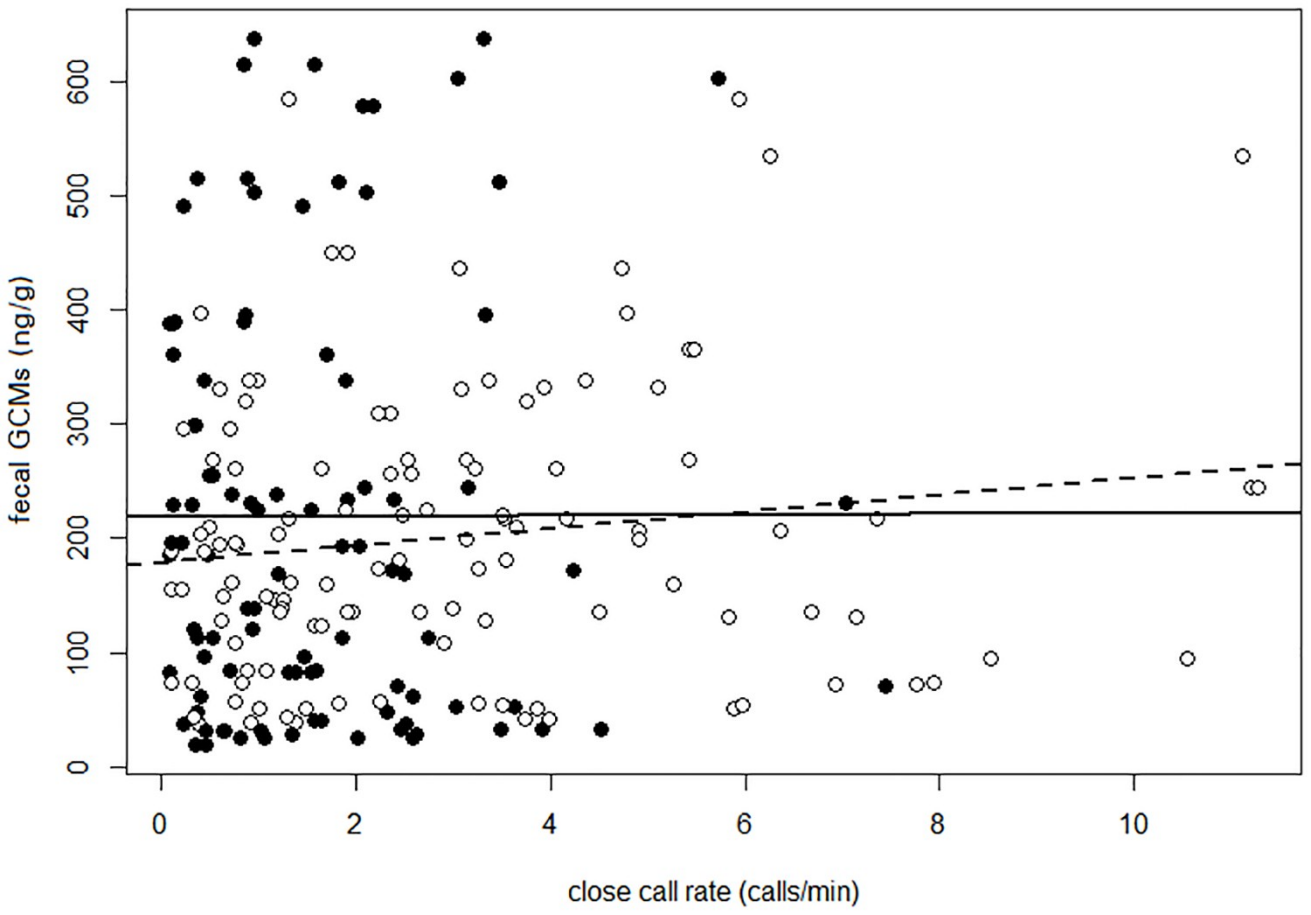
Correlation of individual mean close call rate per session and individual median fecal glucocorticoid metabolite levels per season for the reproductive (black, dashed line) and non-reproductive season (white, black line). (reproductive: t = 1.63, df = 116, P = 0.106, cor = 0.15, non-reproductive: t = 0.03, df = 94, P = 0.978, cor = 0.003).

Close call rate differed significantly between sex, social categories, and season. Specifically, dominant females and one-year old subordinate males called at higher rates than the remaining social classes during the reproductive season (Fig 2a and S2 Table). Additionally, individuals called at significantly higher rates when foraging in windy conditions (Fig 2b and Table 1). Therefore, the model including the interaction between sex, social category, season and wind described call rate patterns the best (Table 1). Fecal GCM concentrations did not have a significant effect on calling rate (Fig 3, *F*_1,32_ = 0.67, *P* = 0.418; Pearson: cor = 0.0585, t = 0.854, df = 212, P = 0.394).

**Table 1:** Minimal model for close call rate as a response variable for both seasons (log-transformed data) (N_rep_ = 66, N_nrep_ = 64).

Explanatory Variable	*numDF*	*denDF*	*F*-value	*P*-value
Social category	3	52	3.18	0.077
Sex	1	55	1.36	0.249
Season	1	52	0.06	0.805
**Wind**	1	128	16.04	**<0.001**
**Social category x sex**	3	55	5.17	**0.003**
**Social category x season**	3	52	2.83	**0.048**
Sex x season	1	52	2.32	0.134
**Social category** x **sex** x **season**	3	52	5.31	**0.003**

Significance is marked with bold font;

x = interaction

To disentangle the three-way interaction, separate models were run for the reproductive (N = 66) and the non-reproductive (N = 64) season. For the reproductive season the model that best described the data included the social category x sex interaction (*F*_3,51_ = 5.70, *P* = 0.002; significant post-hoc tests comparing biologically meaningful social categories: dominant male (DOMM)–dominant female (DOMF): P <0.001; 2 year subordinate male (2M) -1 year subordinate male (1M): P = 0.008; DOMM - 1M: P <0.001). Neither wind (*F*_1,64_ = 2.60, *P* = 0.112) nor lactation state (*F*_1,26_ = 0.13, *P* = 0.717) influenced call rate. For the non-reproductive season neither social category x sex interaction (*F*_3,48_ = 1.12, *P* = 0.350), nor social category (*F*_3,48_ = 1.64, *P* = 0.193) or sex (*F*_1,48_ = 2.29, *P* = 0.137) as main effects were significant explanatory variables of call rate. The external variable wind as a main fixed factor (*F*_12,63_ = 12.36, *P* = 0.001) described the data the best, with higher calling rates recorded in windy conditions. Post-hoc tests are presented in S2 Table.

### Close call structure

**Fig 4:**
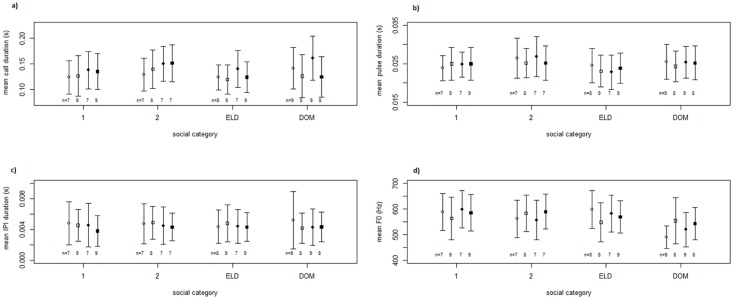
Structural parameters of close calls. a) mean call duration, b) mean pulse duration, c) mean IPI duration, d) mean F0 for the social categories (1: one-year old subordinates, 2: two-year old subordinates, ELD: eldest subordinate, DOM: dominant individuals) for females (○) and males (□) during the reproductive (white) and non-reproductive (black) season. N is the sample size of individuals, ideally 7 calls per individual (for 14 sessions (12 individuals) in the non-reproductive season and 13 sessions (12 individuals) in the reproductive season we only got less than 7 calls/individual (3–6 calls (mainly 5 or 6)) per day were examined. Whiskers show the standard deviation of the data. Graphs show untransformed data. Graphs show untransformed data although the statistical tests were conducted on transformed data and as part of a mixed-model.

The mean values for the acoustic parameters measured of close calls are shown in Table 2. Total call duration was most affected by season, with individuals producing longer calls during the non-reproductive season (*F*_1,58_ = 16.24, *P* < 0.001, Fig 4). Thereby, mainly dominant females produced significantly longer calls during the non-reproductive season resulting in significant sex and social category x sex effects (sex: *F*_1,47_ = 5.94, *P* = 0.019, social category x sex: *F*_3,47_ = 2.88, *P* = 0.046, Fig 4 and Table 3). These effects were not present in the reproductive season.

**Table 2:** Mean values with standard deviation for acoustic parameters analyzed in close calls.

Mean call duration (N_calls_ = 1729)	0.134 +- 0.038 sec
Mean pulse duration (N_calls_ = 1729)	0.025 +- 0.004 sec
Mean IPI duration (N_calls_ = 1727)	0.005 +- 0.002 sec
mean F0 (N_calls_ = 1499)	565.2 +- 77.1 Hz

**Table 3:** Significant posthoc-tests comparing biological meaningful social categories on call structure.

Factor levels tested	Estimate	Std. error	Z-value	P
log(total call duration) ~ social category + sex + season random = ~1|group/caller_ID/season				
non-reproductive season				
social category x sex				
DOM.M—DOM.F = = 0	-0.271	0.075	-3.620	**0.007**
log(F0) ~ social category * sex + season random = ~1 | group/caller_ID/season				
social category				
DOM—1 = = 0	-0.149	0.041	-3.644	**0.002**
DOM—2 = = 0	-0.114	0.041	-2.785	**0.027**
DOM—ELD = = 0	-0.153	0.036	-4.238	**< 0.001**
sex x social category				
DOM.F - 1.F = = 0	-0.149	0.041	-3.641	**0.007**
DOM.F—ELD.F = = 0	-0.150	0.036	-4.124	**< 0.001**
reproductive season				
social category				
DOM—2 = = 0	-0.144	0.047	-3.073	**0.012**
DOM—ELD = = 0	-0.207	0.045	-4.599	**<0.001**
DOM—1 = = 0	-0.181	0.047	-3.877	**<0.001**
social category x sex				
DOM.F - 1.F = = 0	-0.181	0.047	-3.877	**0.003**
DOM.F - 2.F = = 0	-0.144	0.047	-3.073	**0.044**
DOM.F—ELD.F = = 0	-0.207	0.045	-4.599	**< 0.001**
DOM.M—DOM.F = = 0	0.135	0.045	2.990	0.056

Mean pulse duration was best described by the 3-way interaction between social category, sex and season (*F*_3,51_ = 3.84, *P* = 0.015, Fig 4). Mean IPI duration was most affected by season, with calls having shorter IPI durations during the non-reproductive season (*F*_1,58_ = 5.85, *P* = 0.019, Fig 4). The interaction of social category and sex had a significant effect on mean F0, with dominant females having the lowest mean F0, followed by the dominant males (*F*_3,53_ = 2.99, *P* = 0.039, Fig 4 and Table 3 and S2 Table). Social category and season also had significant effects as main factors, with calls having lower mean F0 during the reproductive season (social category: *F*_3,57_ = 5.54, *P* = 0.002; season: *F*_1,57_ = 7.73, *P* = 0.007, Table 3). This pattern was also seen in the separate analysis of the reproductive season (Social category x sex: *F*_3,48_ = 4.74, *P* = 0.006, social category: *F*_3,48_ = 4.69, *P* = 0.006, Table 3). Median weight did not influence any of the structural parameters of the calls (Table 4). Fecal GCMs did not have a significant effect on mean F0 (*F*_1,102_ = 0.82, *P* = 0.366) and were removed from the model. They did not influence any of the structural parameters of the calls (Table 5, S1–S4 Figs).

**Table 4:** P-values of median weight as additional main factor for all tested mixed models (log-transformed) for both seasons together.

Structural parameter	Df	F	P
Mean call duration	127	1.88	0.172
Mean pulse duration	127	2.50	0.117
Mean IPI duration	127	0.98	0.323
Mean F0	122	3.58	0.061

**Table 5:** P-values of fecal glucocorticoid metabolite levels as main factor to all tested mixed models (log transformed) rep = reproductive season, nonrep = non-reproductive season.

Structural parameter	season	Df	F	P
Mean call duration	rep	42	0.0	0.998
Mean pulse duration	rep	42	0.22	0.645
Mean IPI duration	rep	42	0.68	0.413
Mean F0	rep	41	3.52	0.068
Mean call duration	nonrep	46	0.0	0.967
Mean pulse duration	nonrep	46	0.03	0.871
Mean IPI duration	nonrep	46	0.15	0.704
Mean F0	nonrep	45	1.73	0.195

### Factors related to fGCM levels

**Fig 5:**
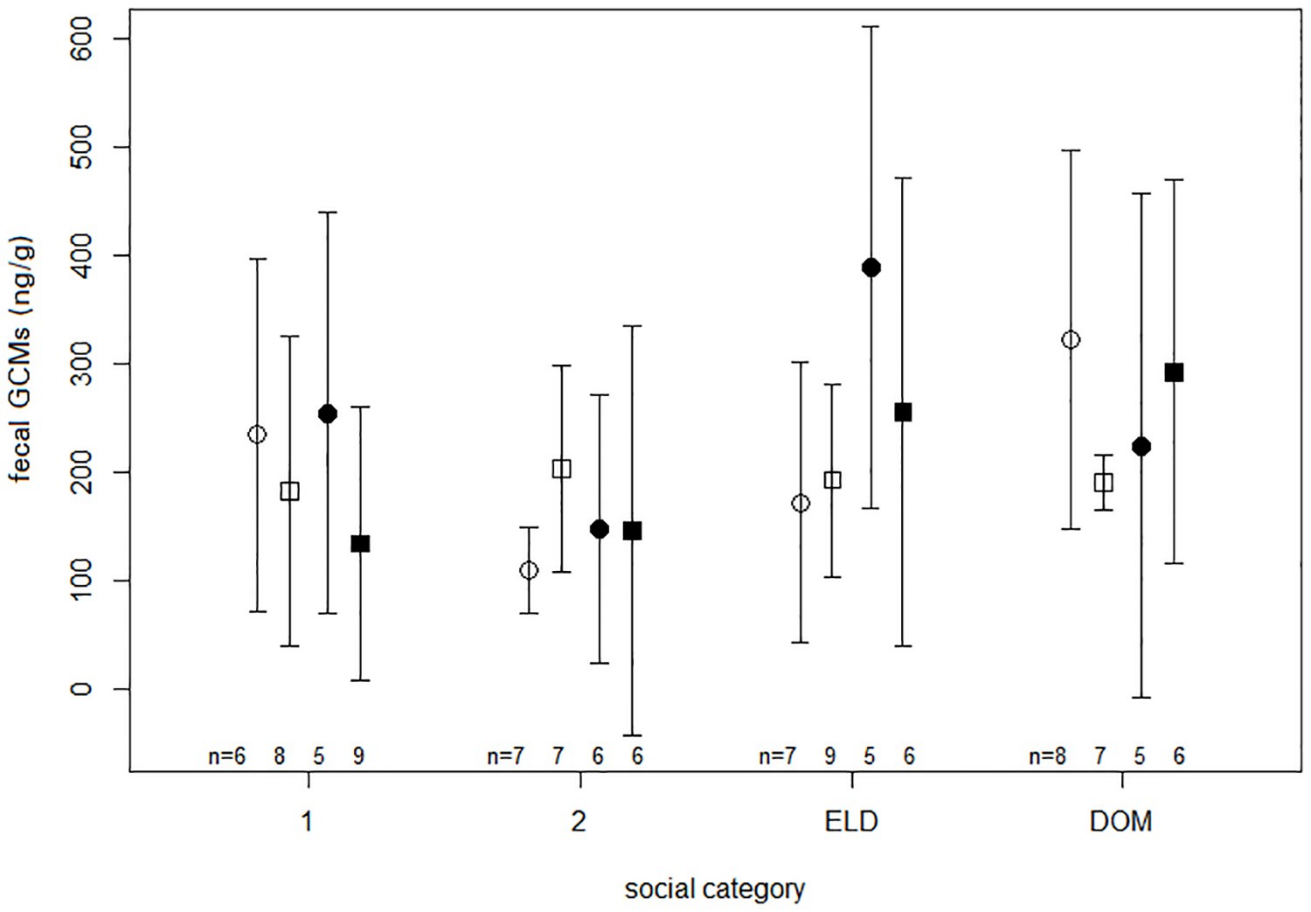
Fecal glucocorticoid metabolite concentrations (ng/g) in the reproductive (white) and non-reproductive (black) season for the different social categories (1, 2, ELD, DOM) and sex (female ○, male □). N: number of focal individual; median glucocorticoid values for each individual were considered for each season; total number samples: Nrep = 154, Nnonrep = 112). Whiskers show the standard deviation of the data. N gives the number of individuals per condition. Graphs show untransformed data.

Fecal GCM concentrations were significantly influenced by social category with dominant individuals having higher fGCM levels than 2-year old subordinates, in a model that included both seasons (Fig 5 and Table 6, significant post-hoc tests: DOM-2 P = 0.012). Season and sex, and their interaction did not have a significant effect on fGCM levels (Table 6). Other tested variables including lactation, access to unrelated mating partners, group size and body weight also had no significant effects on fGCM concentrations. A table with a summary of the collected primary data can be found in the S5 Table.

**Table 6:** Minimal mixed model for fecal glucocorticoid metabolite concentrations as a response variable for the reproductive and non-reproductive season (N_rep_ = 59, N_nrep_ = 48). (log-transformed data)

Explanatory Variable	*numDF*	*denDF*	*F*-value	*P*-value
**Social category**	3	39	3.44	**0.026**
Sex	1	54	0.20	0.658
Season	1	39	0.50	0.484

Significance is marked with bold font.

## Discussion

This study assessed the effects of individual traits, such as sex, age, body weight and social rank, and fGCM concentrations, as well as weather on the close call behavior of foraging meerkats during the reproductive and non-reproductive seasons. Overall, our results reveal a complex pattern of variation in the acoustic parameters measured, associated to the traits considered, suggesting that meerkat close calls potentially provide listeners with cues regarding the producer’s sex, age, rank and reproductive season as well as current weather conditions experienced. Generally, reproductive season had the strongest effect on meerkat close calls, showing associations with call rate, call duration as well as with fGCMs. Fecal GCM concentrations *per se* did not explain significant portions of the variation observed in vocal behavior, however, they correlated positively with social rank independent of season. Close call rate was best explained by the interaction between sex, social category (a composite measure of age and social rank) and season, and was further affected by wind during the reproductive season. Dominant females’ close calls had significantly lower fundamental frequencies than any of the other social categories.

Similar to Japanese macaque contact calls [74], meerkat close calling patterns provided cues with regards to the producer’s sex, age and social rank. Yet, against our expectations, fGCM concentrations did not correlate significantly with tested acoustic parameters. Three, non-mutually exclusive, hypotheses can be put forward to explain our results. First, close calls, which are emitted in relaxed foraging contexts may be more robust against influences by physiological stressors than other call types, such as aggression and alarm calls that have evolved to convey information regarding the producer’s emotional arousal (i.e., higher acoustic specificity, [29, 75]). For example, pig screams, a distress call, better reflect increasing levels of arousal in their acoustic properties compared to grunts, a type of contact calls [75]. Second, we hypothesized that intra-sexual conflict, lactation, breeding and increased foraging competition during the reproductive season [38], would constitute strong chronic stressors to different categories of individuals leading to increases in fGCM levels that would, in turn, be reflected in the individuals’ close calling behavior. However, it is possible that these conditions do not elevate glucocorticoid levels enough or for long enough to affect meerkat vocalizations. Here, we report a maximum 3.5-fold increase within-individual fGCM concentrations between seasons. In contrast, Braga Goncalves and colleagues [33] have reported a 6-fold within-individual increase in fGCM concentrations of an adult subordinate male in response to an intense attack by his group members that resulted in its permanent eviction from the group. Third, it is possible that glucocorticoids *per se* do not directly affect animal vocalizations, although the GC receptors in the vocal tract would suggest otherwise. However, existing literature on the effects of (natural or manipulated) alterations in glucocorticoid levels on vocalizations mostly report effects on distress or alarm calls emitted after the subjects were exposed to intense stressors [29]. Under these conditions, animals mount an acute stress response during which not only glucocorticoids but also catecholamines and neurotransmitters are secreted [76, 77]. Therefore, it remains unclear whether glucocorticoids secreted during the stress response have a direct effect on vocal behavior, or whether they facilitate the action of other components. Further studies are required to tease apart the different components involved in an animal’s stress response and gain a better understanding of the mechanistic links between these components and vocal behavior.

During the reproductive season, dominant females and one-year old males called at the highest rates compared to individuals of other social categories, and also up to 5 times more frequently than during the non-reproductive season. Dominant females may call at higher rates to keep individuals close by, thereby influencing the movement of the group [51, 78, 79] as has been shown in other species [80]. The dominant female takes more frequent leadership during physiologically demanding periods of lactation than in the non-reproductive season where no milk-dependent offspring are to be fed [78]. One-year old males had significantly higher close call rates compared to similarly aged females. Young males and females in many mammalian species differ in their behavioral displays, with males often displaying delayed behavioral maturation (e.g. baboons, *Papio ursinus* [81]), a phenomenon described as infantilism [81, 82]. In general, juvenile meerkats call more frequently compared to adults, which may prevent them from becoming lost from the group [49]. One-year old males may still not show entirely mature behavior and develop more slowly than females of the same age. Hence, the higher call rate in one-year old male meerkats may be a vocal case of male infantilism. Further research should investigate whether 1-year old males also show signs of infantilism in other types of behavior.

Mean pulse duration and mean F0 were mainly influenced by individual traits, namely sex and social category. Dominant females had the lowest F0 compared to all other social categories. With dominance acquisition, female meerkats experience a growth spurt together with endocrine changes such as increased concentrations of androgens [56]. This growth may affect the vocal tract, whereby vocal folds may become elongated, thicker and looser, causing the lower F0 of dominant females [51]. Longer and looser vocal folds could also explain the slightly longer pulse durations of dominant females, since muscles cannot react as fast as more tense vocal folds [51]. However median body weight of individuals did not have an effect on any of the structural parameters of the close calls, which means that overall body mass does not explain the different calling structure of dominant females. Further studies investigating solely anatomical changes in the size and morphology of the vocal tract during aging, instead of the entire body mass as a proxy for overall growth, are needed to verify above mentioned potential changes in vocal tract size.

How individual traits and wind were associated with close call rate and acoustic structure differed between seasons. In the reproductive season close call rate was influenced by the sex, age, and social rank of the producer, while wind had a stronger effect during the non-reproductive season. Furthermore, call duration and IPI duration were mainly influenced by season with longer calls and shorter IPI durations being produced in the non-reproductive season. The general lower calling rate, accompanied by a production of longer calls with the potential for more accurate transmission, may be a compromise between staying in touch with the group while avoiding harassment by juveniles. The higher call rate in windy conditions may be explained by the need of more regular calling in order to maintain group cohesion in noisy environmental conditions [48].

We conclude that close calling patterns in meerkats appear to be influenced by individual traits such as sex, age, and social rank, suggesting a genetically determined and ontogenetic influence, but external factors, such as wind also show an effect. Further studies could enhance our understanding of the influence of age and especially differences between the sexes by investigating anatomical changes of the vocal tract with changes in the acoustic structure of these calls in both males and females. The differences in fGCM concentrations observed between seasons in some of the social categories was not reflected in close calling behavior, suggesting that this call type despite observed acoustic flexibility may be fairly robust to variation in fGCM concentrations, though this requires further information.

## Supporting information

S1 Table Ethogram with recorded activities. All behaviors were mutually exclusive. (With permission adjusted from Engesser, 2011.)
(TIF)

S2 Table P-values for post-hoc Tukey tests run for factors with significant (or significant trend) effects. (PDF)

S3 Table Demographic group composition of 9 sampled groups during the reproductive andnon-reproductive season. Focal individuals are marked in bold. The first 2 individuals listed per group are the dominant individuals. F: Female, M: Male.
(PDF)

S4 Table Sample dates for data collection of focal individuals. (PDF)

S5 Table Individual median close call rate, GCs level, dry fecal weight, mean body weight and close call rate and dry fecal weight range. rep: reproductive season, nonrep: non-reproductive season; SUB: subordinate, DOM: dominant; F: female, M: male.
(PDF)

S6 Table Pearson correlation test for structural close call parameters in relation to fGCM levels. rep = reproductive season, nonrep = non-reproductive season.
(TIF)

S1 Fig Correlation of individual mean close call duration per season, and individual median fGCM level per season. (reproductive (black, dashed line) and non-reproductive season (white, black line)).
(TIF)

S2 Fig Correlation of individual mean pulse close call duration per and individual median fGCM level per season. (reproductive (black, dashed line) and non-reproductive season (white, black line)).
(TIF)

S3 Fig Correlation of individual mean IPI close call duration per and individual median fGCM level per season. (reproductive (black, dashed line) and non-reproductive season (white, black line)).
(TIF)

S4 Fig Correlation of individual mean F0 per and individual median fGCM level per season for the reproductive (black, dashed line) and non-reproductive season (white, black line). (TIF)
