# Supplementary figures and images for: Meerkat close calling patterns are linked to sex, social category, season and wind, but not fecal glucocorticoid metabolite concentrations
Source: PLoS One. 2017 May 3;12(5):e0175371. doi: 10.1371/journal.pone.0175371 (PMC5414979; doi:10.1371/journal.pone.0175371)

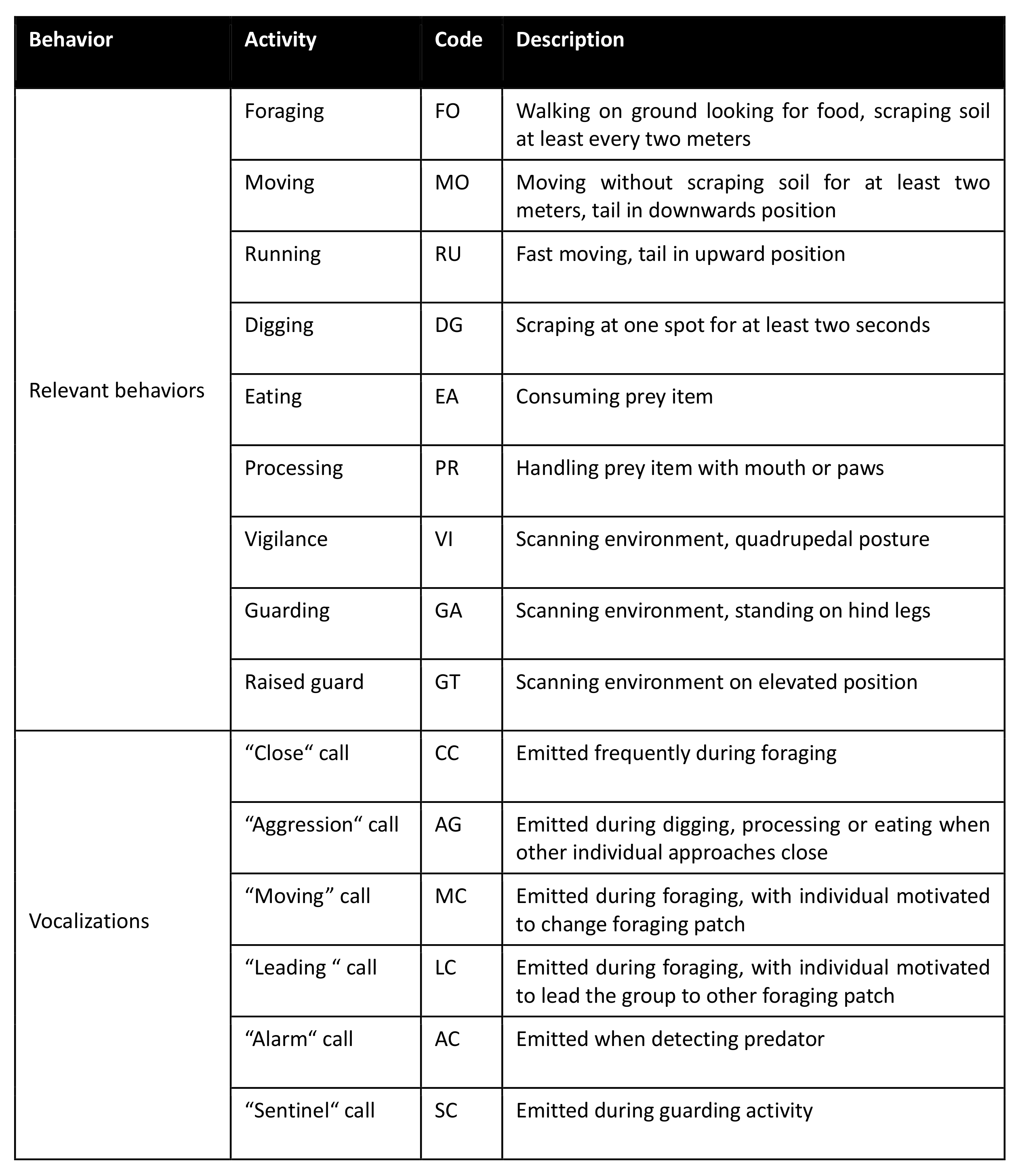

Supplement: S1 Table — All behaviors were mutually exclusive. (With permission adjusted from Engesser, 2011.) (TIF) [file pone.0175371.s001.tif]

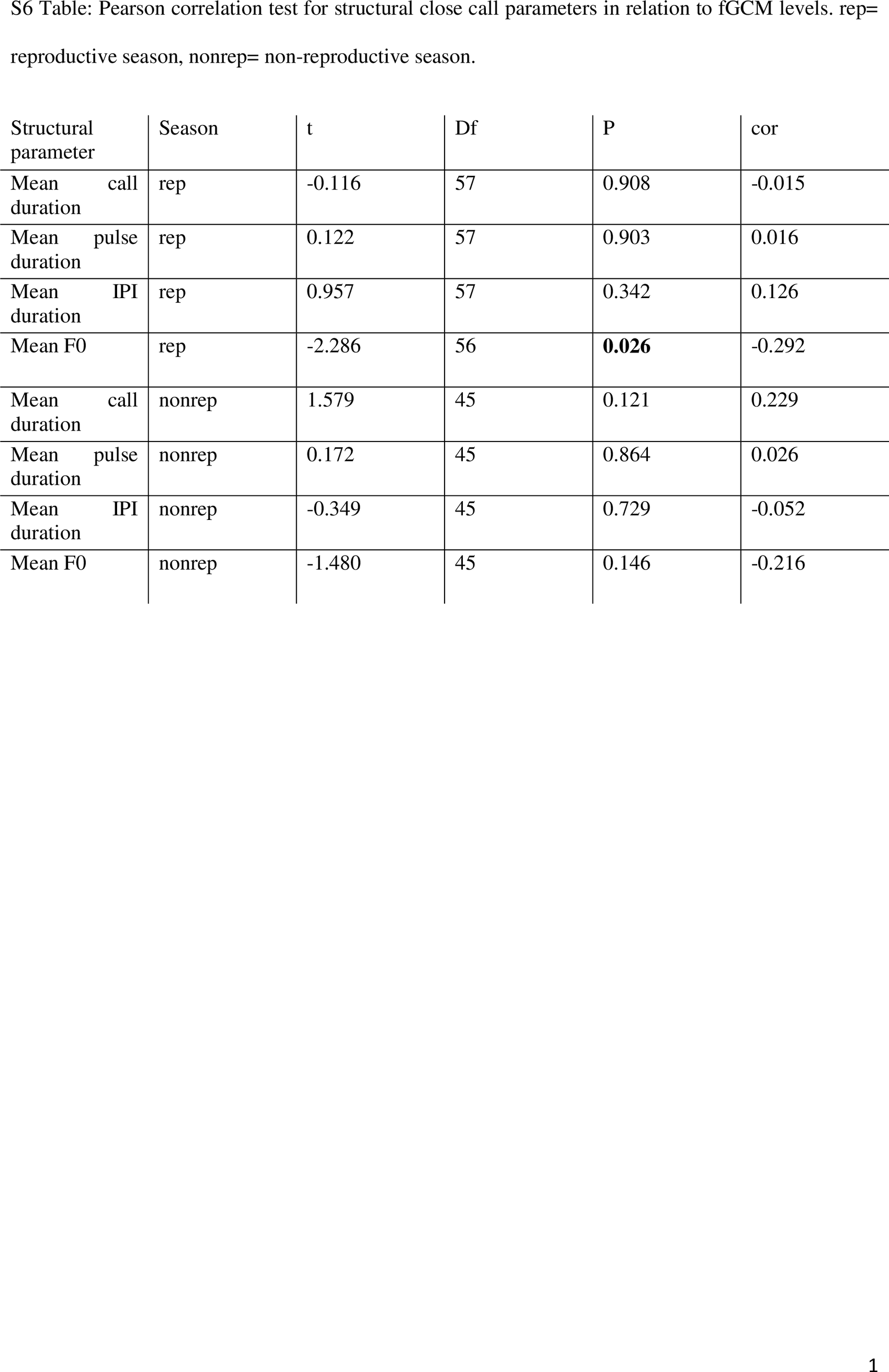

Supplement: S6 Table — rep = reproductive season, nonrep = non-reproductive season. (TIF) [file pone.0175371.s006.tif]

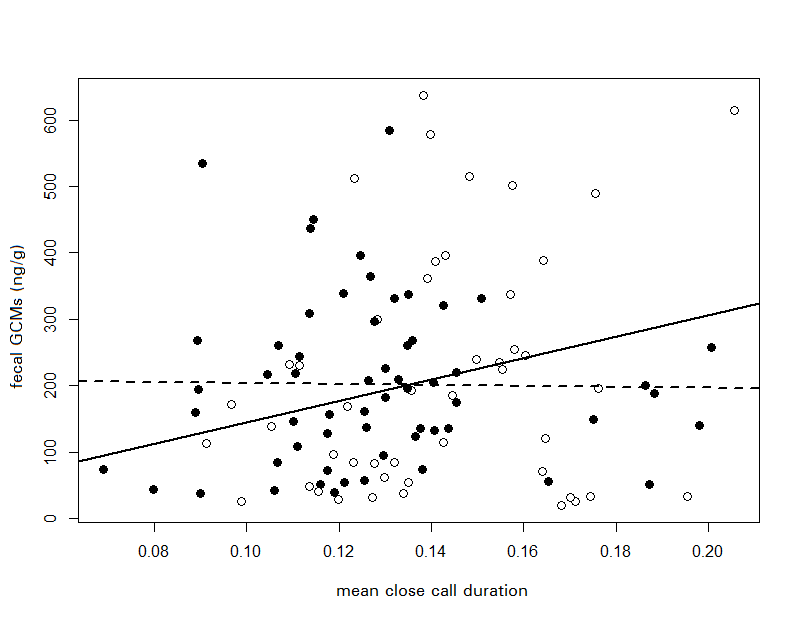

Supplement: S1 Fig — (reproductive (black, dashed line) and non-reproductive season (white, black line)). (TIF) [file pone.0175371.s007.tif]

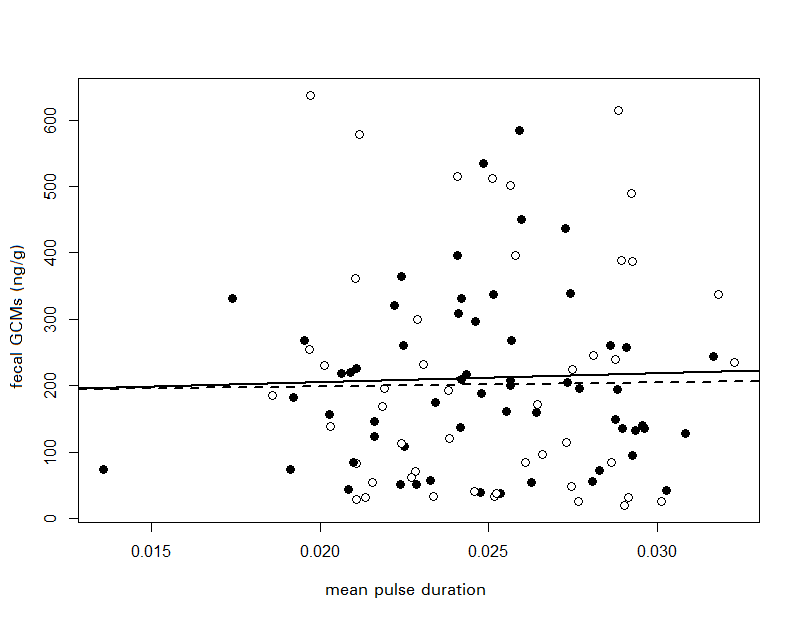

Supplement: S2 Fig — (reproductive (black, dashed line) and non-reproductive season (white, black line)). (TIF) [file pone.0175371.s008.tif]

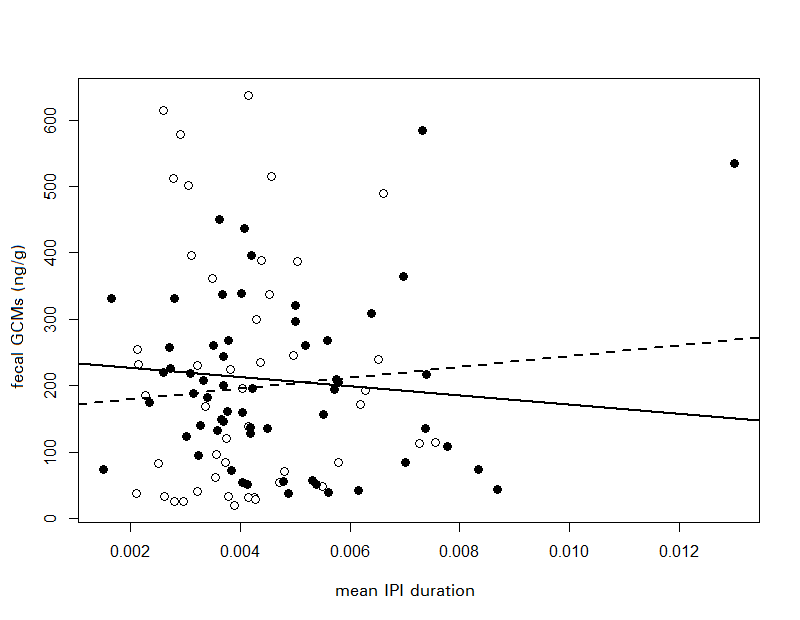

Supplement: S3 Fig — (reproductive (black, dashed line) and non-reproductive season (white, black line)). (TIF) [file pone.0175371.s009.tif]

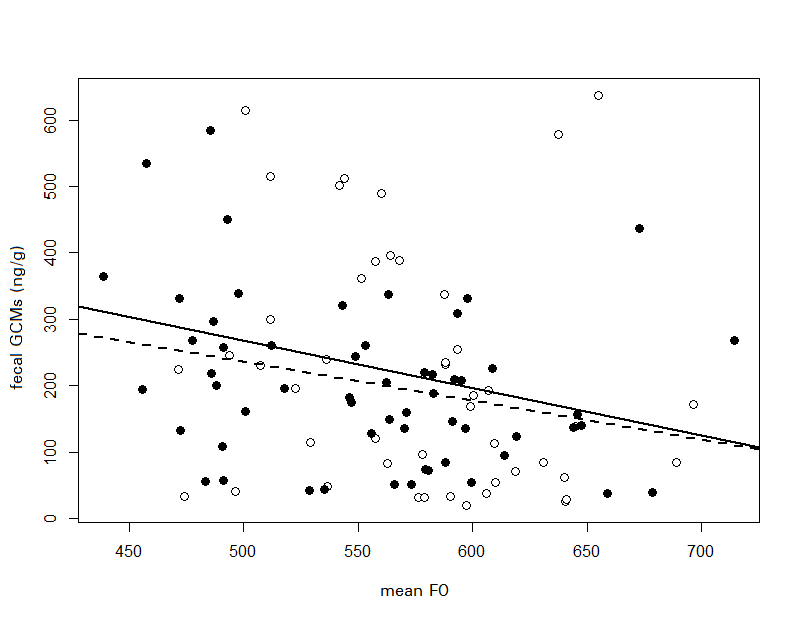

Supplement: S4 Fig — (TIF) [file pone.0175371.s010.tif]
