# Supplementary material for: Meerkat close calling patterns are linked to sex, social category, season and wind, but not fecal glucocorticoid metabolite concentrations
Source: PLoS One. 2017 May 3;12(5):e0175371. doi: 10.1371/journal.pone.0175371 (PMC5414979; doi:10.1371/journal.pone.0175371)
Supplement: S2 Table — (PDF) [file pone.0175371.s002.pdf]

**Factor levels tested**	***Estimate***	***Std. error***	***Z-value***	***P***
**log(CC rate) ~ social category * sex * season + wind random = ~1 | group/ID/season**				
**social category**				
DOM - 2 == 0	1.02224	0.37962	2.693	0.0356
**social category x season**				
2.rep - 1.rep == 0	- 0.9461161	0.3158555	-2.995	0.05516
2.nonrep - 1.rep == 0	-1.1523795	0.3241604	-3.555	0.00903
ELD.nonrep - 1.rep == 0	-1.1514481	0.3131687	-3.677	0.00574
DOM.nonrep - 1.rep == 0	-1.1278799	0.3043452	-3.706	0.00509
**social category x sex x season**				
2.M.rep - DOM.F.rep == 0	-1.334316	0.403406	-3.308	0.0719
DOM.M.rep - DOM.F.rep == 0	-1.589626	0.391357	-4.062	<0.01
2.F.nonrep - DOM.F.rep == 0	-1.754606	0.418308	-4.195	<0.01
ELD.F.nonrep - DOM.F.rep == 0	-1.719547	0.418188	-4.112	<0.01
DOM.F.nonrep - DOM.F.rep == 0	-1.616728	0.375914	-4.301	<0.01
DOM.M.rep - 1.M.rep == 0	-1.521164	0.391429	-3.886	<0.01
2.F.nonrep - 1.M.rep == 0	-1.686144	0.418376	-4.030	<0.01
ELD.F.nonrep - 1.M.rep == 0	-1.651085	0.418267	-3.947	<0.01

DOM.F.nonrep - 1.M.rep == 0	-1.548266	0.391286	-3.957	<0.01
**reproductive season**				
**social category**				
DOM - 2 == 0	0.9981	0.3464	2.881	0.0203
**social category x sex**				
DOM.M - 1.F == 0	-1.09742	0.37484	-2.928	0.06673
2.M - DOM.F == 0	-1.36225	0.36020	-3.782	0.00370
ELD.M - DOM.F == 0	-1.05299	0.34848	-3.022	0.05120
DOM.M - DOM.F == 0	-1.59074	0.34848	-4.565	< 0.001
2.M - 1.M == 0	-1.29267	0.36020	-3.589	0.00795
ELD.M - 1.M == 0	-0.98342	0.34848	-2.822	0.08908
DOM.M - 1.M == 0	-1.52116	0.34848	-4.365	< 0.001
**log(total call duration) ~ social category + sex + season random = ~1|group/caller_ID/season**				
**non-reproductive season**				
**social category x sex**				
ELD.M - DOM.F == 0	-0.24837	0.07238	-3.431	0.0136
DOM.M - DOM.F == 0	-0.27105	0.07488	-3.620	0.0069
**log(F0) ~ social category * sex + season random = ~1 | group/caller_ID/season**				

**social category**				
DOM - 1 == 0	-0.149104	0.040922	-3.644	0.00153
DOM - 2 == 0	-0.113964	0.040922	-2.785	0.02736
DOM - ELD == 0	-0.153026	0.036109	-4.238	< 0.001
**sex x social category**				
F.DOM - F.1 == 0	-0.148500	0.040788	-3.641	0.00661
F.DOM - M.1 == 0	-0.119445	0.037814	-3.159	0.03382
F.DOM - F.ELD == 0	-0.150255	0.036430	-4.124	< 0.001
F.DOM - M.2 == 0	-0.147225	0.039387	-3.738	.00464
**reproductive season**				
**social category**				
DOM - 2 == 0	-0.14371	0.04677	-3.073	0.0116
DOM - ELD == 0	-0.20710	0.04503	-4.599	<0.001
DOM - 1 == 0	-0.18134	0.04677	-3.877	<0.001
**social category x sex**				
DOM.F - 1.F == 0	-0.181338	0.046771	-3.877	0.00259
DOM.F - 2.F == 0	-0.143712	0.046771	-3.073	0.04390
DOM.F - ELD.F == 0	-0.207098	0.045030	-4.599	< 0.001

DOM.M - DOM.F == 0	0.134671	0.045034	2.990	0.05625
1.M - DOM.F == 0	0.141985	0.043631	3.254	0.02542
2.M - DOM.F == 0	0.186636	0.045030	4.145	< 0.001
**log(glucocorticoids) ~ social category + sex + season random = ~1 | group/ID**				
**social category**				
DOM - 2 == 0	0.6850	0.2236	3.063	0.0117
