# Supplementary material for: Meerkat close calling patterns are linked to sex, social category, season and wind, but not fecal glucocorticoid metabolite concentrations
Source: PLoS One. 2017 May 3;12(5):e0175371. doi: 10.1371/journal.pone.0175371 (PMC5414979; doi:10.1371/journal.pone.0175371)
Supplement: S3 Table — Focal individuals are marked in bold. The first 2 individuals listed per group are the dominant individuals. F: Female, M: Male. (PDF) [file pone.0175371.s003.pdf]

S3 Table:
Demographic group composition of 9 sampled groups during the reproductive and non-reproductive season. Focal individuals are marked in bold.
The first 2 individuals listed per group are the dominant individuals. F:Female, M: Male.

Reproductive Season

**GRP_1**	**37 members**	
Date of birth	ID	Sex
16.09.2004	**ID_64**	F
NA	**ID_4**	M
25.01.2008	**ID_5**	M
25.01.2008	M	M
26.11.2008	M	M
22.02.2009	M	M
22.02.2009	**ID_1**	F
19.09.2009	M	M
19.09.2009	M	M
03.10.2009	**ID_6**	M
03.10.2009	**ID_2**	F
12.12.2009	M	M
26.02.2010	F	F
08.11.2010	M	M
08.11.2010	M	M
08.11.2010	**ID_7**	F
08.11.2010	**ID_3**	M
27.01.2011	M	M
27.01.2011	M	M
27.01.2011	M	M
27.01.2011	F	F
27.01.2011	M	M
27.01.2011	M	M
27.01.2011	M	M
06.09.2011	M	M
06.09.2011	M	M
06.09.2011	M	M
06.09.2011	F	F
06.09.2011	M	M
23.11.2011	F	F
23.11.2011	M	M
23.11.2011	M	M
12.02.2012	PUP	PUP
12.02.2012	PUP	PUP
12.02.2012	PUP	PUP
12.02.2012	PUP	PUP
12.02.2012	PUP	PUP

**GRP_2**	**22 members**	
Date of birth	ID	Sex
30.01.2005	**ID_9**	F

Non-reproductive Season

**GRP_1**	**37 members**	
Date of birth	ID	Sex
16.09.2004	**ID_64**	F
NA	**ID_4**	M
25.01.2008	**ID_5**	M
25.01.2008	M	M
26.11.2008	M	M
22.02.2009	M	M
22.02.2009	**ID_1**	F
19.09.2009	M	M
19.09.2009	M	M
03.10.2009	**ID_6**	M
03.10.2009	**ID_2**	F
12.12.2009	M	M
26.02.2010	F	F
08.11.2010	M	M
08.11.2010	M	M
08.11.2010	**ID_7**	F
08.11.2010	**ID_3**	M
27.01.2011	M	M
27.01.2011	M	M
27.01.2011	M	M
27.01.2011	F	F
27.01.2011	M	M
27.01.2011	M	M
27.01.2011	M	M
06.09.2011	M	M
06.09.2011	M	M
06.09.2011	M	M
06.09.2011	F	F
06.09.2011	M	M
23.11.2011	F	F
23.11.2011	M	M
23.11.2011	M	M
12.02.2012	PUP	PUP
12.02.2012	PUP	PUP
12.02.2012	PUP	PUP
12.02.2012	PUP	PUP
12.02.2012	PUP	PUP

**GRP_2**	**22 members**	
Date of birth	ID	Sex
30.01.2005	**ID_9**	F

NA	**ID_8**	M
01.12.2008	**ID_49**	F
01.12.2008	**ID_51**	M
20.09.2009	F	F
20.09.2009	F	F
20.09.2009	M	M
20.09.2009	M	M
08.12.2009	M	M
08.12.2009	**ID_50**	F
08.12.2009	**ID_52**	M
02.03.2010	M	M
02.03.2010	M	M
24.08.2010	M	M
24.08.2010	M	M
29.01.2011	**ID_53**	M
01.08.2011	F	F
09.01.2012	M	M
27.01.2012	M	M
27.01.2012	M	M
27.01.2012	F	F
27.01.2012	M	M

GRP_3 15 members

Date of birth	ID	Sex
03.09.2006	**ID_10**	F
18.09.2008	**ID_14**	M
29.11.2009	**ID_16**	M
22.02.2010	F	F
22.02.2010	**ID_18**	M
22.02.2010	**ID_11**	F
24.08.2010	F	F
09.12.2010	M	M
28.02.2011	**ID_19**	M
28.02.2011	**ID_12**	F
28.02.2011	F	F
28.02.2011	M	M
15.02.2012	PUP	PUP
15.02.2012	PUP	PUP
15.02.2012	PUP	PUP

GRP_4 16 members

Date of birth	ID	Sex
08.12.2007	**ID_20**	F
07.03.2008	**ID_13**	M
18.08.2008	F	F
30.12.2008	**ID_15**	M
22.01.2009	**ID_21**	F
15.09.2009	F	F
22.02.2010	**ID_17**	M
26.03.2010	**ID_22**	F

NA	**ID_8**	M
01.12.2008	**ID_49**	F
01.12.2008	**ID_51**	M
20.09.2009	F	F
20.09.2009	F	F
20.09.2009	M	M
20.09.2009	M	M
08.12.2009	M	M
08.12.2009	**ID_50**	F
08.12.2009	**ID_52**	M
02.03.2010	M	M
02.03.2010	M	M
24.08.2010	M	M
24.08.2010	M	M
29.01.2011	**ID_53**	M
01.08.2011	F	F
09.01.2012	M	M
27.01.2012	M	M
27.01.2012	M	M
27.01.2012	F	F
27.01.2012	M	M

GRP_3 15 members

Date of birth	ID	Sex
03.09.2006	**ID_10**	F
18.09.2008	**ID_14**	M
29.11.2009	**ID_16**	M
22.02.2010	F	F
22.02.2010	**ID_18**	M
22.02.2010	**ID_11**	F
24.08.2010	F	F
09.12.2010	M	M
28.02.2011	**ID_19**	M
28.02.2011	**ID_12**	F
28.02.2011	F	F
28.02.2011	M	M
15.02.2012	PUP	PUP
15.02.2012	PUP	PUP
15.02.2012	PUP	PUP

GRP_4 15 members

Date of birth	ID	Sex
08.12.2007	**ID_20**	F
07.03.2008	**ID_13**	M
18.08.2008	F	F
30.12.2008	**ID_15**	M
22.01.2009	**ID_21**	F
15.09.2009	F	F
22.02.2010	**ID_17**	M
26.03.2010	**ID_22**	F

24.08.2010	M	M
26.08.2010	M	M
30.01.2011	**ID_24**	M
30.01.2011	**ID_23**	F
23.01.2012	M	M
23.01.2012	M	M
23.01.2012	F	F
23.01.2012	M	M

**GRP_5**	**23 members**	
Date of birth	ID	Sex
04.12.2004	**ID_41**	F
13.08.2008	**ID_29**	M
18.01.2009	M	M
18.01.2009	**ID_26**	F
19.09.2009	F	F
19.09.2009	F	F
13.12.2009	**ID_27**	F
13.12.2009	**ID_33**	M
18.08.2010	M	M
18.08.2010	M	M
17.11.2010	**ID_28**	F
17.11.2010	**ID_34**	M
17.11.2010	F	F
07.02.2011	M	M
07.02.2011	M	M
07.02.2011	F	F
07.02.2011	M	M
07.02.2011	M	M
07.08.2011	M	M
16.01.2012	PUP	PUP
16.01.2012	PUP	PUP
16.01.2012	PUP	PUP
16.01.2012	PUP	PUP

**GRP_6**	**33 members**	
Date of birth	ID	Sex
23.11.2003	**ID_25**	F
06.12.2004	**ID_66**	M
09.11.2008	**ID_38**	M
09.11.2008	**ID_35**	F
30.03.2009	M	M
14.09.2009	M	M
05.12.2009	**ID_39**	M
05.12.2009	**ID_36**	F
05.12.2009	M	M
05.12.2009	F	F
21.02.2010	F	F
21.02.2010	M	M
10.08.2010	M	M

24.08.2010	M	M
26.08.2010	M	M
30.01.2011	**ID_24**	M
30.01.2011	**ID_23**	F
23.01.2012	M	M
23.01.2012	M	M
23.01.2012	F	F

**GRP_5**	**22 members**	
Date of birth	ID	Sex
04.12.2004	**ID_41**	F
13.08.2008	**ID_29**	M
18.01.2009	**ID_26**	F
19.09.2009	F	F
19.09.2009	F	F
13.12.2009	**ID_27**	F
13.12.2009	**ID_33**	M
18.08.2010	M	M
18.08.2010	M	M
17.11.2010	**ID_28**	F
17.11.2010	**ID_34**	M
17.11.2010	F	F
07.02.2011	M	M
07.02.2011	M	M
07.02.2011	F	F
07.02.2011	M	M
07.02.2011	M	M
07.08.2011	M	M
16.01.2012	PUP	PUP
16.01.2012	PUP	PUP
16.01.2012	PUP	PUP
16.01.2012	PUP	PUP

**GRP_6**	**32 members**	
Date of birth	ID	Sex
23.11.2003	**ID_25**	F
06.12.2004	**ID_66**	M
09.11.2008	**ID_38**	M
09.11.2008	**ID_35**	F
30.03.2009	M	M
14.09.2009	M	M
05.12.2009	**ID_39**	M
05.12.2009	**ID_36**	F
05.12.2009	M	M
05.12.2009	F	F
21.02.2010	M	M
10.08.2010	M	M
10.08.2010	F	F

10.08.2010	F	F
10.08.2010	F	F
28.10.2010	M	M
28.10.2010	F	F
28.10.2010	F	F
17.01.2011	**ID_40**	M
17.01.2011	M	M
17.01.2011	**ID_37**	F
17.01.2011	M	M
16.03.2011	F	F
16.03.2011	M	M
23.04.2011	M	M
23.04.2011	M	M
23.04.2011	F	F
14.08.2011	F	F
14.08.2011	F	F
14.08.2011	M	M
14.08.2011	F	F
27.12.2011	F	F
14.01.2012	PUP	PUP

**GRP_7**	**30 members**	
Date of birth	ID	Sex
12.03.2005	**ID_42**	F
25.01.2007	**ID_67**	M
04.01.2008	**ID_43**	F
24.08.2008	**ID_46**	M
24.08.2008	F	F
24.08.2008	F	F
11.11.2008	M	M
29.01.2009	M	M
29.01.2009	F	F
12.09.2009	F	F
24.09.2009	F	F
24.09.2009	F	F
24.09.2009	F	F
04.12.2009	F	F
04.12.2009	**ID_47**	M
04.12.2009	**ID_44**	F
21.02.2010	M	M
04.11.2010	M	M
04.11.2010	M	M
04.11.2010	M	M
22.01.2011	**ID_48**	M
22.01.2011	**ID_45**	F
22.01.2011	M	M
22.01.2011	F	F
22.01.2011	F	F
08.05.2011	F	F
08.05.2011	M	M

10.08.2010	F	F
28.10.2010	M	M
28.10.2010	F	F
28.10.2010	F	F
17.01.2011	**ID_40**	M
17.01.2011	M	M
17.01.2011	**ID_37**	F
17.01.2011	M	M
16.03.2011	F	F
16.03.2011	M	M
23.04.2011	M	M
23.04.2011	M	M
23.04.2011	F	F
14.08.2011	F	F
14.08.2011	F	F
14.08.2011	M	M
14.08.2011	F	F
27.12.2011	F	F
14.01.2012	PUP	PUP

**GRP_7**	**33 members**	
Date of birth	ID	Sex
12.03.2005	**ID_42**	F
25.01.2007	**ID_67**	M
04.01.2008	**ID_43**	F
24.08.2008	**ID_46**	M
24.08.2008	F	F
24.08.2008	F	F
11.11.2008	M	M
29.01.2009	M	M
29.01.2009	F	F
12.09.2009	F	F
24.09.2009	F	F
24.09.2009	F	F
24.09.2009	F	F
04.12.2009	F	F
04.12.2009	**ID_47**	M
04.12.2009	**ID_44**	F
21.02.2010	M	M
04.11.2010	M	M
04.11.2010	M	M
04.11.2010	M	M
22.01.2011	**ID_48**	M
22.01.2011	**ID_45**	F
22.01.2011	M	M
22.01.2011	F	F
22.01.2011	F	F
08.05.2011	F	F
08.05.2011	M	M

08.05.2011	M	M
08.05.2011	M	M
31.08.2011	M	M

**GRP_8**	**10 members**	
Date of birth	ID	Sex
03.09.2010	**ID_55**	F
31.10.2008	**ID_31**	M
31.10.2008	**ID_30**	M
18.01.2009	M	M
13.12.2009	**ID_32**	M
28.02.2010	**ID_54**	F
21.01.2011	**ID_56**	M
08.01.2012	F	F
08.01.2012	M	M
08.01.2012	M	M

**GRP_9**	**35 members**	
Date of birth	ID	Sex
26.08.2005	**ID_65**	F
NA	**ID_60**	M
07.04.2008	**ID_61**	M
16.08.2008	**ID_57**	F
21.11.2009	M	M
21.11.2009	M	M
21.11.2009	M	M
09.02.2010	**ID_58**	F
09.02.2010	M	M
09.02.2010	F	F
09.02.2010	**ID_62**	**M**
30.04.2010	M	M
30.04.2010	F	F
30.04.2010	F	F
30.04.2010	F	F
22.09.2010	F	F
22.09.2010	F	F
22.09.2010	M	M
06.12.2010	**ID_63**	**M**
06.12.2010	M	M
06.12.2010	**ID_59**	**F**
06.12.2010	M	M
28.02.2011	M	M
28.02.2011	M	M
28.02.2011	F	F
15.08.2011	M	M
15.08.2011	M	M
02.11.2011	M	M

08.05.2011	M	M
08.05.2011	M	M
31.08.2011	M	M
26.02.2012	M	M
26.02.2012	F	F
26.02.2012	F	F

**GRP_8**	**9 members**	
Date of birth	ID	Sex
28.02.2010	**ID_54**	F
31.10.2008	**ID_31**	M
31.10.2008	**ID_30**	M
18.01.2009	M	M
13.12.2009	**ID_32**	M
21.01.2011	**ID_56**	M
08.01.2012	F	F
08.01.2012	M	M
08.01.2012	M	M

**GRP_9**	**32 members**	
Date of birth	ID	Sex
26.08.2005	**ID_65**	F
NA	**ID_60**	M
07.04.2008	**ID_61**	M
16.08.2008	**ID_57**	F
21.11.2009	M	M
21.11.2009	M	M
21.11.2009	M	M
09.02.2010	**ID_58**	F
09.02.2010	M	M
09.02.2010	F	F
09.02.2010	**ID_62**	**M**
30.04.2010	M	M
30.04.2010	F	F
30.04.2010	F	F
30.04.2010	F	F
22.09.2010	F	F
22.09.2010	F	F
22.09.2010	M	M
06.12.2010	**ID_63**	**M**
06.12.2010	M	M
06.12.2010	**ID_59**	**F**
06.12.2010	M	M
28.02.2011	M	M
28.02.2011	M	M
28.02.2011	F	F
15.08.2011	M	M
15.08.2011	M	M
02.11.2011	M	M

02.11.2011	M	M
02.11.2011	M	M
26.01.2012	M	M
26.01.2012	F	F
26.01.2012	F	F
26.01.2012	M	M
26.01.2012	M	M

02.11.2011	M	M
02.11.2011	M	M
26.01.2012	M	M
26.01.2012	F	F
