# Supplementary material for: Meerkat close calling patterns are linked to sex, social category, season and wind, but not fecal glucocorticoid metabolite concentrations
Source: PLoS One. 2017 May 3;12(5):e0175371. doi: 10.1371/journal.pone.0175371 (PMC5414979; doi:10.1371/journal.pone.0175371)
Supplement: S4 Table — (PDF) [file pone.0175371.s004.pdf]

S4 Table: Individual sampling dates and time for close call behavior, close call recordings and feces collection. rep: reproductive season, nonrep: non-reproductive season; SUB: subordinate, DOM: dominant; F: female, M: male

identity	season	group	social	status	sex	age	date rate data	date rate data	time rate data	time rate data	date structure data	date structure data	time structure data	time structure data	sampling	sampling
			class				1st session	2nd session	1st session	2nd session	1st session	2nd session	1st session	2nd session	dates feces	time feces
ID_1	rep	GRP_1	ELD	SUB	F	3	03.03.2012	08.03.2012	09:06:22	09:35:19	03.03.2012	08.03.2012	am	am	03.03.2012	09:00:00
ID_2	rep	GRP_1	2	SUB	F	2	29.02.2012	08.03.2012	09:15:04	09:49:04	29.02.2012	08.03.2012	am	am	03.04.2012	17:59:00
ID_3	rep	GRP_1	1	SUB	F	1	29.02.2012	08.03.2012	08:51:01	09:02:02	29.02.2012	08.03.2012	am	am	no	no
ID_4	rep	GRP_1	DOM	DOM	M	NA	03.03.2012	08.03.2012	09:49:20	10:15:25	no	no	no	no	no	no
ID_5	rep	GRP_1	ELD	SUB	M	3	29.02.2012	13.03.2012	09:01:50	07:35:06	29.02.2012	08.03.2012	am	am	09.05.2012	16:50:00
ID_6	rep	GRP_1	2	SUB	M	2	29.02.2012	08.03.2012	09:29:31	09:24:37	29.02.2012	08.03.2012	am	am	03.04.2012	10:15:00
															14.04.2012	17:32:00
ID_7	rep	GRP_1	1	SUB	M	1	03.03.2012	08.03.2012	09:36:37	09:13:37	29.02.2012	08.03.2012	am	am	no	no
ID_8	rep	GRP_2	DOM	DOM	M	NA	04.02.2012	08.02.2012	13:09:10	09:33:10	01.02.2012	08.02.2012	07:40:00	08:32:00	23.03.2012	09:23:00
ID_9	rep	GRP_2	DOM	DOM	F	7	04.02.2012	08.02.2012	12:42:32	09:17:54	01.02.2012	08.02.2012	07:03:00	08:40:00	08.02.2012	08:10:00
															25.03.2012	08:02:00
															26.04.2012	18:00:00
ID_10	rep	GRP_3	DOM	DOM	F	5	04.03.2012	11.03.2012	07:50:37	09:51:30	04.03.2012	11.03.2012	08:36:00	07:40:00	11.03.2012	10:03:00
															11.03.2012	07:50:00
															14.03.2012	09:25:00
															29.03.2012	17:30:00
															24.03.2012	07:41:00
ID_11	rep	GRP_3	ELD	SUB	F	2	04.03.2012	11.03.2012	08:24:33	09:39:16	04.03.2012	11.03.2012	08:50:00	08:05:00	09.03.2012	10:49:00
															29.03.2012	17:32:00
ID_12	rep	GRP_3	1	SUB	F	1	04.03.2012	11.03.2012	08:36:39	09:15:05	04.03.2012	11.03.2012	08:00:00	08:30:00	15.03.2012	18:25:00
															23.03.2012	09:43:00
															24.03.2012	06:38:00
															13.04.2012	10:08:00
ID_13	rep	GRP_4	DOM	DOM	M	4	04.02.2012	11.02.2012	10:18:55	08:52:30	04.02.2012	11.02.2012	08:06:00	11:50:00	05.03.2012	18:15:00
															01.04.2012	18:30:00
ID_14	rep	GRP_3	DOM	DOM	M	3	04.03.2012	11.03.2012	08:51:15	09:03:51	04.03.2012	11.03.2012	08:10:00	08:37:00	07.03.2012	09:55:00
															14.03.2012	09:25:00
															29.03.2012	17:25:00
															06.04.2012	17:41:00
ID_15	rep	GRP_4	ELD	SUB	M	3	09.02.2012	11.02.2012	08:08:44	09:28:37	04.02.2012	11.02.2012	07:30:00	11:40:00	21.02.2012	09:58:00
															08.04.2012	17:50:00
ID_16	rep	GRP_3	ELD	SUB	M	2	04.03.2012	11.03.2012	09:05:08	08:52:41	04.03.2012	11.03.2012	08:20:00	08:15:00	07.03.2012	10:35:00
															14.03.2012	09:25:00
ID_17	rep	GRP_4	2	SUB	M	2	04.02.2012	12.02.2012	10:03:42	11:05:01	04.02.2012	12.02.2012	09:45:00	am	04.02.2012	10:10:00
															24.03.2012	18:21:00
															28.03.2012	10:30:00
															29.03.2012	16:39:00
ID_18	rep	GRP_3	2	SUB	M	2	04.03.2012	11.03.2012	08:13:42	10:04:39	04.03.2012	11.03.2012	09:06:00	07:55:00	07.03.2012	09:45:00
															14.03.2012	09:25:00
															15.03.2012	18:55:00
															13.04.2012	10:02:00

ID_19	rep	GRP_3	1	SUB	M	1	04.03.2012	11.03.2012	08:01:51	09:27:52	04.03.2012	11.03.2012	08:30:00	08:23:00	07.03.2012	08:00:00
															23.03.2012	18:06:00
															06.04.2012	17:41:00
															02.04.2012	07:54:00
ID_20	rep	GRP_4	DOM	DOM	F	4	04.02.2012	11.02.2012	10:50:08	08:18:12	04.02.2012	11.02.2012	08:20:00	10:05:00	23.02.2012	09:42:00
															26.03.2012	16:39:00
ID_21	rep	GRP_4	ELD	SUB	F	3	04.02.2012	11.02.2012	10:38:40	08:29:44	04.02.2012	11.02.2012	08:45:00	11:10:00	08.03.2012	10:57:00
															13.03.2012	18:45:00
ID_22	rep	GRP_4	2	SUB	F	2	04.02.2012	11.02.2012	11:01:10	08:05:12	04.02.2012	11.02.2012	08:40:00	10:50:00	23.02.2012	19:09:00
															13.03.2012	18:41:00
															23.03.2012	09:51:00
															24.03.2012	18:40:00
															09.04.2012	09:43:00
ID_23	rep	GRP_4	1	SUB	F	1	04.02.2012	11.02.2012	09:24:53	09:16:25	04.02.2012	11.02.2012	07:40:00	10:20:00	19.02.2012	09:01:00
ID_24	rep	GRP_4	1	SUB	M	1	04.02.2012	11.02.2012	09:10:14	09:04:26	04.02.2012	11.02.2012	07:55:00	10:45:00	04.02.2012	10:30:00
															28.02.2012	09:36:00
															18.03.2012	09:28:00
															02.05.2012	16:59:00
ID_25	rep	GRP_6	DOM	DOM	F	8	02.02.2012	05.02.2012	08:53:30	09:50:19	31.01.2012	05.02.2012	08:30:00	07:54:00	31.03.2012	16:54:00
															02.04.2012	17:50:00
ID_26	rep	GRP_5	ELD	SUB	F	3	03.02.2012	10.02.2012	10:48:30	12:07:01	03.02.2012	10.02.2012	08:55:00	10:40:00	10.04.2012	10:47:00
ID_27	rep	GRP_5	2	SUB	F	2	03.02.2012	10.02.2012	11:02:44	11:49:27	03.02.2012	10.02.2012	09:15:00	09:00:00	03.02.2012	11:02:00
															07.03.2012	18:30:00
															19.03.2012	07:58:00
															10.04.2012	08:52:00
ID_28	rep	GRP_5	1	SUB	F	1	03.02.2012	16.02.2012	12:32:22	10:30:49	03.02.2012	16.02.2012	12:30:00	10:25:00	20.02.2012	07:45:00
															28.02.2012	18:36:00
ID_29	rep	GRP_5	DOM	DOM	M	3	03.02.2012	10.02.2012	10:34:47	12:23:24	03.02.2012	10.02.2012	13:15:00	10:45:00	10.02.2012	10:30:00
															28.02.2012	18:00:00
															29.03.2012	09:52:00
ID_30	rep	GRP_8	ELD	SUB	M	3	02.02.2012	06.02.2012	10:39:34	08:59:24	06.02.2012	30.01.2012	07:25:00	06:50:00	30.01.2012	09:25:00
															28.02.2012	09:26:00
															06.03.2012	18:51:00
ID_31	rep	GRP_8	DOM	DOM	M	3	02.02.2012	06.02.2012	11:02:45	08:03:23	06.02.2012	30.01.2012	07:07:00	08:05:00	30.01.2012	09:25:00
															06.03.2012	18:50:00
															13.03.2012	18:40:00
															16.03.2012	08:33:00
ID_32	rep	GRP_8	2	SUB	M	2	02.02.2012	09.02.2012	10:27:59	07:28:28	30.01.2012	06.02.2012	08:20:00	07:15:00	13.03.2012	18:40:00
ID_33	rep	GRP_5	ELD	SUB	M	2	03.02.2012	10.02.2012	11:43:31	11:06:06	10.02.2012	03.02.2012	10:20:00	10:32:00	10.03.2012	08:05:00
															17.03.2012	09:44:00
ID_34	rep	GRP_5	1	SUB	M	1	03.02.2012	10.02.2012	11:19:10	11:38:57	03.02.2012	15.02.2012	13:00:00	10:10:00	20.02.2012	07:45:00
															17.04.2012	11:45:00
ID_36	rep	GRP_6	2	SUB	F	2	02.02.2012	05.02.2012	08:23:37	09:26:24	31.01.2012	05.02.2012	07:55:00	08:30:00	31.01.2012	07:56:00
															02.04.2012	17:12:00

ID_37	rep	GRP_6	1	SUB	F	1	02.02.2012	05.02.2012	07:59:05	10:07:39	31.01.2012	05.02.2012	09:50:00	08:45:00	31.01.2012	09:50:00
															05.02.2012	10:25:00
ID_38	rep	GRP_6	ELD	SUB	M	3	02.02.2012	05.02.2012	07:47:35	09:11:14	31.01.2012	05.02.2012	10:30:00	09:00:00	05.02.2012	10:05:00
															15.02.2012	08:45:00
ID_39	rep	GRP_6	2	SUB	M	2	02.02.2012	05.02.2012	08:35:46	10:33:45	31.01.2012	05.02.2012	07:20:00	08:00:00	31.01.2012	07:37:00
															18.03.2012	10:15:00
ID_40	rep	GRP_6	1	SUB	M	1	02.02.2012	05.02.2012	08:10:49	10:22:30	31.01.2012	05.02.2012	07:30:00	08:40:00	31.01.2012	07:35:00
															02.04.2012	18:32:00
															18.04.2012	09:30:00
ID_41	rep	GRP_5	DOM	DOM	F	7	03.02.2012	10.02.2012	11:31:17	11:18:44	03.02.2012	10.02.2012	12:05:00	09:15:00	20.03.2012	17:50:00
															18.04.2012	17:21:00
ID_42	rep	GRP_7	DOM	DOM	F	7	05.03.2012	12.03.2012	09:18:31	10:05:47	05.03.2012	12.03.2012	08:15:00	08:39:00	09.05.2012	17:18:00
															24.03.2012	17:25:00
ID_43	rep	GRP_7	ELD	SUB	F	4	05.03.2012	12.03.2012	10:08:07	09:31:25	05.03.2012	12.03.2012	08:53:00	07:50:00	04.05.2012	16:55:00
ID_44	rep	GRP_7	2	SUB	F	2	05.03.2012	12.03.2012	10:25:15	09:13:03	05.03.2012	12.03.2012	08:40:00	08:02:00	20.03.2012	08:40:00
ID_45	rep	GRP_7	1	SUB	F	1	05.03.2012	12.03.2012	09:07:04	10:18:41	05.03.2012	12.03.2012	08:30:00	07:56:00	20.03.2012	09:50:00
															09.05.2012	17:21:00
ID_46	rep	GRP_7	ELD	SUB	M	3	06.03.2012	13.03.2012	08:46:38	09:38:23	06.03.2012	13.03.2012	08:30:00	09:20:00	04.05.2012	16:45:00
ID_47	rep	GRP_7	2	SUB	M	2	05.03.2012	12.03.2012	10:36:50	09:00:11	05.03.2012	12.03.2012	08:00:00	08:50:00	no	no
ID_48	rep	GRP_7	1	SUB	M	1	05.03.2012	12.03.2012	09:40:25	09:42:55	05.03.2012	12.03.2012	08:07:00	08:21:00	15.03.2012	08:35:00
															26.03.2012	10:52:00
															17.04.2012	08:55:00
															22.04.2012	11:05:00
															16.05.2012	08:58:00
ID_49	rep	GRP_2	ELD	SUB	F	3	08.02.2012	13.02.2012	10:42:47	08:02:36	13.02.2012	08.02.2012	07:55:00	07:20:00	06.04.2012	18:06:00
ID_50	rep	GRP_2	2	SUB	F	2	04.02.2012	08.02.2012	12:08:14	10:08:45	01.02.2012	08.02.2012	07:53:00	08:25:00	08.02.2012	09:55:00
															15.02.2012	18:19:00
															26.04.2012	18:00:00
															24.04.2012	16:05:00
ID_51	rep	GRP_2	ELD	SUB	M	3	04.02.2012	08.02.2012	12:54:42	09:54:51	01.02.2012	08.02.2012	07:24:00	07:30:00	08.02.2012	08:10:00
															14.04.2012	11:02:00
ID_52	rep	GRP_2	2	SUB	M	2	04.02.2012	08.02.2012	12:31:32	10:22:38	01.02.2012	08.02.2012	07:57:00	08:20:00	25.03.2012	10:40:00
															02.04.2012	08:20:00
ID_53	rep	GRP_2	1	SUB	M	1	04.02.2012	08.02.2012	13:22:23	09:03:41	01.02.2012	08.02.2012	07:35:00	08:10:00	04.02.2012	13:30:00
															08.02.2012	08:55:00
															13.02.2012	08:01:00
															22.03.2012	17:33:00
															25.03.2012	10:23:00
															14.04.2012	11:02:00
ID_54	rep	GRP_8	ELD	SUB	F	2	02.02.2012	06.02.2012	10:03:37	08:16:36	06.02.2012	30.01.2012	07:40:00	07:35:00	30.01.2012	07:35:00
															02.04.2012	09:51:00
ID_55	rep	GRP_8	DOM	DOM	F	1	02.02.2012	06.02.2012	10:51:08	08:27:41	06.02.2012	30.01.2012	07:00:00	06:58:00	30.01.2012	09:30:00
															06.02.2012	08:00:00
															18.02.2012	08:39:00
															13.03.2012	18:40:00

ID_56	rep	GRP_8	1	SUB	M	1	02.02.2012	06.02.2012	10:16:10	08:39:04	06.02.2012	30.01.2012	08:00:00	07:58:00	30.01.2012	09:25:00
															02.02.2012	11:20:00
															23.02.2012	19:00:00
ID_57	rep	GRP_9	ELD	SUB	F	3	07.02.2012	14.02.2012	10:14:25	10:26:59	07.02.2012	14.02.2012	08:25:00	09:10:00	no	no
ID_58	rep	GRP_9	2	SUB	F	2	14.02.2012	16.02.2012	10:42:24	08:43:25	07.02.2012	14.02.2012	08:04:00	08:40:00	04.04.2012	09:55:00
ID_59	rep	GRP_9	1	SUB	F	1	07.02.2012	16.02.2012	11:02:19	09:06:42	07.02.2012	16.02.2012	08:40:00	08:15:00	07.02.2012	11:20:00
															29.03.2012	09:30:00
															18.04.2012	17:34:00
ID_60	rep	GRP_9	DOM	DOM	M	NA	07.02.2012	14.02.2012	09:59:12	10:10:24	07.02.2012	14.02.2012	08:50:00	08:25:00	no	no
ID_61	rep	GRP_9	ELD	SUB	M	4	07.02.2012	14.02.2012	11:25:45	09:22:21	07.02.2012	14.02.2012	07:50:00	08:50:00	25.04.2012	17:36:00
ID_62	rep	GRP_9	2	SUB	M	2	07.02.2012	16.02.2012	10:32:26	08:27:55	07.02.2012	14.02.2012	09:15:00	09:00:00	04.04.2012	09:58:00
ID_63	rep	GRP_9	1	SUB	M	1	07.02.2012	14.02.2012	10:47:11	09:35:51	07.02.2012	14.02.2012	09:30:00	08:20:00	07.02.2012	11:05:00
															24.02.2012	18:05:00
															18.04.2012	17:37:00
															07.02.2012	11:50:00
ID_64	rep	GRP_1	DOM	DOM	F	7	03.03.2012	08.03.2012	09:21:08	10:02:35	29.02.2012	08.03.2012	am	am	17.04.2012	17:55:00
ID_65	rep	GRP_9	DOM	DOM	F	7	07.02.2012	14.02.2012	09:48:01	09:54:38	07.02.2012	14.02.2012	08:10:00	08:10:00	no	no
ID_66	rep	GRP_6	DOM	DOM	M	7	02.02.2012	05.02.2012	09:13:59	09:37:35	31.01.2012	05.02.2012	08:06:00	08:15:00	19.04.2012	10:28:00
ID_67	rep	GRP_7	DOM	DOM	M	5	05.03.2012	12.03.2012	09:29:32	09:54:17	05.03.2012	12.03.2012	08:23:00	08:16:00	20.03.2012	11:05:00
															22.04.2012	17:40:00
															09.05.2012	17:21:00
															18.05.2012	10:51:00
ID_1	nonrep	GRP_1	ELD	SUB	F	3	15.06.2012	22.06.2012	10:38:45	12:29:30	17.06.2012	22.06.2012	14:40:00	11:10:00	08.06.2012	16:00:00
															14.06.2012	15:25:00
ID_2	nonrep	GRP_1	2	SUB	F	2	15.06.2012	22.06.2012	09:27:44	11:53:22	15.06.2012	22.06.2012	NA	NA	30.05.2012	10:20:00
															26.06.2012	12:10:00
ID_3	nonrep	GRP_1	1	SUB	F	1	15.06.2012	22.06.2012	10:04:57	13:19:26	15.06.2012	22.06.2012	NA	11:35:00	no	no
ID_4	nonrep	GRP_1	DOM	DOM	M	NA	15.06.2012	22.06.2012	11:06:19	13:07:50	no	no	no	no	26.06.2012	12:10:00
ID_5	nonrep	GRP_1	ELD	SUB	M	4	15.06.2012	22.06.2012	10:52:55	12:17:14	15.06.2012	22.06.2012	11:50:00	10:20:00	no	no
ID_6	nonrep	GRP_1	2	SUB	M	2	15.06.2012	22.06.2012	10:16:04	12:55:50	15.06.2012	22.06.2012	12:03:00	11:15:00	no	no
ID_7	nonrep	GRP_1	1	SUB	M	1	15.06.2012	22.06.2012	09:38:43	12:39:27	15.06.2012	22.06.2012	11:40:00	10:50:00	26.06.2012	12:10:00
ID_8	nonrep	GRP_2	DOM	DOM	M	NA	05.05.2012	12.05.2012	10:29:40	11:27:42	05.05.2012	12.05.2012	08:25:00	09:30:00	no	no
ID_9	nonrep	GRP_2	DOM	DOM	F	7	05.05.2012	12.05.2012	09:44:53	12:11:58	05.05.2012	12.05.2012	08:35:00	09:10:00	no	no
ID_10	nonrep	GRP_3	DOM	DOM	F	5	16.06.2012	23.06.2012	10:25:50	10:55:15	16.06.2012	23.06.2012	10:39:00	11:10:00	22.06.2012	16:35:00
															26.06.2012	16:52:00
ID_11	nonrep	GRP_3	ELD	SUB	F	2	16.06.2012	23.06.2012	10:37:35	11:25:09	16.06.2012	23.06.2012	11:12:00	10:00:00	no	no
ID_12	nonrep	GRP_3	1	SUB	F	1	16.06.2012	23.06.2012	11:01:03	11:50:14	16.06.2012	23.06.2012	10:15:00	11:05:00	04.06.2012	16:32:00
															11.06.2012	15:55:00
															13.06.2012	15:50:00
ID_13	nonrep	GRP_4	DOM	DOM	M	4	14.05.2012	21.05.2012	12:31:47	12:59:59	14.05.2012	21.05.2012	10:50:00	11:15:00	10.06.2012	16:50:00
ID_14	nonrep	GRP_3	DOM	DOM	M	3	16.06.2012	23.06.2012	11:23:30	10:30:18	16.06.2012	23.06.2012	10:30:00	11:35:00	20.06.2012	16:45:00
															22.06.2012	16:20:00
															26.06.2012	16:52:00

ID_15	nonrep	GRP_4	ELD	SUB	M	3	14.05.2012	21.05.2012	12:20:53	12:49:59	14.05.2012	21.05.2012	11:50:00	10:15:00	14.05.2012	12:50:00
															30.05.2012	17:00:00
															10.06.2012	17:25:00
ID_16	nonrep	GRP_3	ELD	SUB	M	2	16.06.2012	23.06.2012	10:14:16	11:37:56	16.06.2012	23.06.2012	11:00:00	11:50:00	13.06.2012	14:30:00
															20.06.2012	16:45:00
															20.06.2012	16:45:00
ID_18	nonrep	GRP_3	2	SUB	M	2	16.06.2012	23.06.2012	11:37:09	11:07:17	16.06.2012	23.06.2012	11:22:00	11:24:00	04.06.2012	17:35:00
															20.06.2012	16:45:00
ID_19	nonrep	GRP_3	1	SUB	M	1	16.06.2012	23.06.2012	11:11:55	10:41:33	16.06.2012	23.06.2012	11:36:00	10:20:00	25.06.2012	15:50:00
ID_20	nonrep	GRP_4	DOM	DOM	F	4	14.05.2012	21.05.2012	13:14:57	12:04:10	14.05.2012	21.05.2012	11:10:00	11:00:00	24.05.2012	17:32:00
															30.05.2012	16:10:00
															10.06.2012	16:45:00
ID_21	nonrep	GRP_4	ELD	SUB	F	3	14.05.2012	21.05.2012	12:53:34	12:38:25	14.05.2012	21.05.2012	10:30:00	10:40:00	28.05.2012	17:40:00
ID_22	nonrep	GRP_4	2	SUB	F	2	14.05.2012	21.05.2012	12:08:37	12:28:25	14.05.2012	21.05.2012	12:00:00	10:25:00	14.05.2012	12:15:00
															18.05.2012	10:30:00
															30.05.2012	17:25:00
															10.06.2012	17:05:00
ID_23	nonrep	GRP_4	1	SUB	F	1	14.05.2012	21.05.2012	12:43:32	11:48:14	14.05.2012	21.05.2012	10:50:00	10:45:00	17.05.2012	16:47:00
															28.05.2012	16:40:00
ID_24	nonrep	GRP_4	1	SUB	M	1	14.05.2012	21.05.2012	13:04:03	12:13:49	14.05.2012	21.05.2012	12:07:00	11:35:00	14.05.2012	10:55:00
															21.05.2012	12:20:00
															28.05.2012	17:30:00
															06.06.2012	17:15:00
ID_25	nonrep	GRP_6	DOM	DOM	F	8	02.05.2012	09.05.2012	11:16:23	12:29:49	02.05.2012	09.05.2012	10:15:00	11:10:00	no	no
ID_26	nonrep	GRP_5	ELD	SUB	F	3	28.05.2012	05.06.2012	11:15:03	11:14:22	28.05.2012	05.06.2012	09:40:00	10:46:00	29.05.2012	16:47:00
ID_27	nonrep	GRP_5	2	SUB	F	2	28.05.2012	05.06.2012	11:26:32	10:17:56	28.05.2012	05.06.2012	11:05:00	09:58:00	no	no
ID_28	nonrep	GRP_5	1	SUB	F	1	28.05.2012	05.06.2012	11:03:01	10:28:30	28.05.2012	05.06.2012	10:03:00	10:15:00	28.05.2012	12:45:00
															05.06.2012	10:05:00
															05.06.2012	12:40:00
															23.06.2012	16:35:00
ID_29	nonrep	GRP_5	DOM	DOM	M	3	28.05.2012	05.06.2012	10:29:41	10:07:03	28.05.2012	05.06.2012	11:09:00	09:35:00	14.05.2012	07:59:00
															05.06.2012	10:05:00
ID_30	nonrep	GRP_8	ELD	SUB	M	3	23.04.2012	01.05.2012	11:11:27	10:45:43	23.04.2012	01.05.2012	10:00:00	10:25:00	07.05.2012	09:09:00
															07.05.2012	17:49:00
															12.05.2012	17:17:00
															17.05.2012	10:33:00
ID_31	nonrep	GRP_8	DOM	DOM	M	3	23.04.2012	01.05.2012	10:22:36	11:18:37	23.04.2012	01.05.2012	NA	09:45:00	23.04.2012	17:47:00
															07.05.2012	09:09:00
															07.05.2012	17:48:00
															12.05.2012	17:06:00
ID_32	nonrep	GRP_8	2	SUB	M	2	23.04.2012	01.05.2012	10:34:51	11:33:30	01.05.2012	23.04.2012	NA	NA	07.05.2012	16:50:00
ID_33	nonrep	GRP_5	ELD	SUB	M	2	28.05.2012	05.06.2012	10:40:11	11:02:12	05.06.2012	28.05.2012	10:25:00	10:48:00	05.06.2012	13:00:00
															05.06.2012	13:00:00
ID_34	nonrep	GRP_5	1	SUB	M	1	28.05.2012	05.06.2012	10:18:49	10:39:25	28.05.2012	05.06.2012	09:50:00	10:07:00	31.05.2012	16:15:00

ID_35	nonrep	GRP_6	ELD	SUB	F	3	02.05.2012	09.05.2012	12:06:53	11:40:31	02.05.2012	09.05.2012	09:20:00	11:10:00	no	no
ID_36	nonrep	GRP_6	2	SUB	F	2	02.05.2012	09.05.2012	11:39:51	12:18:26	02.05.2012	09.05.2012	09:50:00	10:50:00	05.05.2012	17:45:00
ID_37	nonrep	GRP_6	1	SUB	F	1	02.05.2012	09.05.2012	12:29:54	09:20:45	02.05.2012	09.05.2012	10:20:00	09:40:00	11.05.2012	16:47:00
															17.05.2012	17:25:00
ID_38	nonrep	GRP_6	ELD	SUB	M	3	02.05.2012	09.05.2012	11:27:42	09:43:10	02.05.2012	09.05.2012	10:30:00	09:50:00	05.06.2012	16:50:00
ID_39	nonrep	GRP_6	2	SUB	M	2	02.05.2012	09.05.2012	10:54:50	12:06:41	02.05.2012	09.05.2012	09:45:00	10:45:00	17.05.2012	17:22:00
ID_40	nonrep	GRP_6	1	SUB	M	1	02.05.2012	09.05.2012	11:50:53	11:17:44	02.05.2012	09.05.2012	09:30:00	11:00:00	07.05.2012	17:43:00
															29.05.2012	16:10:00
															05.06.2012	16:55:00
ID_41	nonrep	GRP_5	DOM	DOM	F	7	28.05.2012	05.06.2012	10:52:00	10:51:28	28.05.2012	05.06.2012	10:30:00	10:36:00	no	no
ID_42	nonrep	GRP_7	DOM	DOM	F	7	18.06.2012	27.06.2012	10:01:12	10:19:50	18.06.2012	27.06.2012	09:40:00	10:44:00	18.06.2012	16:46:00
ID_43	nonrep	GRP_7	ELD	SUB	F	4	18.06.2012	27.06.2012	11:08:29	09:55:48	18.06.2012	27.06.2012	09:58:00	09:34:00	15.06.2012	16:20:00
ID_44	nonrep	GRP_7	2	SUB	F	2	18.06.2012	27.06.2012	09:51:01	10:41:14	18.06.2012	27.06.2012	NA	11:19:00	18.06.2012	16:37:00
															22.06.2012	15:25:00
ID_45	nonrep	GRP_7	1	SUB	F	1	18.06.2012	27.06.2012	10:23:41	10:53:10	18.06.2012	27.06.2012	10:37:00	10:34:00	no	no
ID_46	nonrep	GRP_7	ELD	SUB	M	3	18.06.2012	30.06.2012	11:33:12	09:57:05	18.06.2012	30.06.2012	11:45:00	09:25:00	18.06.2012	11:15:00
ID_47	nonrep	GRP_7	2	SUB	M	2	18.06.2012	27.06.2012	10:48:22	09:44:49	18.06.2012	27.06.2012	10:35:00	09:55:00	18.06.2012	11:05:00
															22.06.2012	15:30:00
ID_48	nonrep	GRP_7	1	SUB	M	1	18.06.2012	27.06.2012	10:12:48	10:08:19	18.06.2012	27.06.2012	10:57:00	11:21:00	18.05.2012	10:17:00
															22.06.2012	15:25:00
															26.06.2012	12:15:00
ID_49	nonrep	GRP_2	ELD	SUB	F	3	05.05.2012	12.05.2012	10:42:12	11:17:42	05.05.2012	12.05.2012	NA	NA	08.05.2012	07:47:00
															12.05.2012	09:20:00
ID_50	nonrep	GRP_2	2	SUB	F	2	05.05.2012	12.05.2012	10:53:58	10:37:08	05.05.2012	12.05.2012	09:45:00	08:20:00	08.05.2012	17:44:00
															26.05.2012	17:20:00
															25.05.2012	17:29:00
ID_51	nonrep	GRP_2	ELD	SUB	M	3	05.05.2012	12.05.2012	09:56:19	12:23:56	05.05.2012	12.05.2012	09:25:00	NA	18.05.2012	17:45:00
ID_52	nonrep	GRP_2	2	SUB	M	2	05.05.2012	12.05.2012	10:18:34	11:38:22	05.05.2012	12.05.2012	09:15:00	NA	08.05.2012	07:55:00
															18.05.2012	17:50:00
ID_53	nonrep	GRP_2	1	SUB	M	1	05.05.2012	12.05.2012	10:07:22	12:01:25	05.05.2012	12.05.2012	08:45:00	09:00:00	08.05.2012	09:15:00
															10.05.2012	17:27:00
															15.05.2012	10:13:00
															26.05.2012	10:50:00
ID_54	nonrep	GRP_8	DOM	DOM	F	2	23.04.2012	01.05.2012	10:49:48	10:57:26	23.04.2012	01.05.2012	09:55:00	NA	23.04.2012	17:45:00
															07.05.2012	09:33:00
															07.05.2012	16:50:00
															12.05.2012	17:32:00
															26.05.2012	09:56:00
ID_56	nonrep	GRP_8	1	SUB	M	1	23.04.2012	01.05.2012	11:00:23	11:08:01	23.04.2012	01.05.2012	10:10:00	10:00:00	07.05.2012	09:46:00
															12.05.2012	17:19:00
															18.05.2012	16:26:00
															28.05.2012	16:55:00
ID_58	nonrep	GRP_9	2	SUB	F	2	31.05.2012	07.06.2012	09:51:44	10:52:08	31.05.2012	07.06.2012	10:45:00	10:18:00	no	no

ID_59	nonrep	GRP_9	1	SUB	F	1	31.05.2012	07.06.2012	10:02:38	11:36:21	31.05.2012	07.06.2012	10:50:00	10:05:00	04.05.2012	11:25:00
															23.05.2012	16:30:00
															12.06.2012	16:10:00
ID_60	nonrep	GRP_9	DOM	DOM	M	NA	31.05.2012	07.06.2012	10:27:00	10:23:25	31.05.2012	07.06.2012	11:30:00	10:38:00	no	no
ID_61	nonrep	GRP_9	ELD	SUB	M	4	31.05.2012	07.06.2012	10:16:16	10:38:40	31.05.2012	07.06.2012	10:25:00	10:28:00	no	no
ID_62	nonrep	GRP_9	2	SUB	M	2	31.05.2012	07.06.2012	09:34:29	11:04:31	31.05.2012	07.06.2012	09:15:00	11:39:00	23.05.2012	17:10:00
ID_63	nonrep	GRP_9	1	SUB	M	1	31.05.2012	07.06.2012	10:41:14	10:10:35	31.05.2012	07.06.2012	09:45:00	11:00:00	23.05.2012	16:00:00
															31.05.2012	10:45:00
															27.05.2012	16:05:00
ID_64	nonrep	GRP_1	DOM	DOM	F	7	15.06.2012	22.06.2012	10:27:12	12:06:27	15.06.2012	22.06.2012	12:20:00	10:40:00	26.06.2012	12:10:00
ID_65	nonrep	GRP_9	DOM	DOM	F	6	31.05.2012	07.06.2012	09:16:43	11:25:39	31.05.2012	07.06.2012	08:30:00	10:50:00	08.05.2012	17:48:00
ID_66	nonrep	GRP_6	DOM	DOM	M	7	02.05.2012	09.05.2012	11:06:11	11:55:00	02.05.2012	09.05.2012	10:05:00	10:25:00	no	no
ID_67	nonrep	GRP_7	DOM	DOM	M	5	18.06.2012	27.06.2012	10:35:12	10:31:45	18.06.2012	27.06.2012	09:30:00	10:18:00	11.06.2012	17:20:00
															14.06.2012	17:04:00
															18.06.2012	11:50:00
															22.06.2012	15:25:00
