# Supplementary material for: Meerkat close calling patterns are linked to sex, social category, season and wind, but not fecal glucocorticoid metabolite concentrations
Source: PLoS One. 2017 May 3;12(5):e0175371. doi: 10.1371/journal.pone.0175371 (PMC5414979; doi:10.1371/journal.pone.0175371)
Supplement: S5 Table — rep: reproductive season, nonrep: non-reproductive season; SUB: subordinate, DOM: dominant; F: female, M: male. (PDF) [file pone.0175371.s005.pdf]

S5 Table: Individual median close call rate, GCs level, dry fecal weight, mean body weight and close call rate and dry fecal weight range. rep: reproductive season, nonrep: non-reproductive season; SUB: subordinate, DOM: dominant; F: female, M: male

identity	season	group	access unrelated mating partners	social class	status	sex	age	median close call rate	close call rate minimum	cloce call rate maximum	median GCs (ng/g)	mean weight (g)	median dry fecal weight	min dry fecal weight	max dry fecal weight
ID_1	rep	GRP_1	NO	ELD	SUB	F	3	1,613	1,579	1,647	122,970	674,000	0,101	NA	NA
ID_2	rep	GRP_1	NO	2	SUB	F	2	0,302	0,226	0,379	37,059	612,500	0,102	NA	NA
ID_3	rep	GRP_1	NO	1	SUB	F	1	1,450	0,541	2,359	NA	559,000	no	no	no
ID_4	rep	GRP_1	YES	DOM	DOM	M	NA	1,004	0,808	1,200	NA	NA	no	no	no
ID_5	rep	GRP_1	NO	ELD	SUB	M	3	0,203	0,101	0,306	73,636	685,333	0,110	NA	NA
ID_6	rep	GRP_1	NO	2	SUB	M	2	0,944	0,899	0,989	338,036	662,250	0,107	0,103	0,110
ID_7	rep	GRP_1	NO	1	SUB	M	1	4,605	2,955	6,256	NA	542,500	no	no	no
ID_8	rep	GRP_2	YES	DOM	DOM	M	NA	0,692	0,604	0,781	194,257	825,000	0,101	NA	NA
ID_9	rep	GRP_2	YES	DOM	DOM	F	7	5,447	5,418	5,475	365,047	795,500	0,105	0,101	0,107
ID_10	rep	GRP_3	NO	DOM	DOM	F	5	8,691	6,263	11,119	534,857	609,000	0,101	0,100	0,105
ID_11	rep	GRP_3	NO	ELD	SUB	F	2	2,412	1,300	3,523	217,190	583,000	0,104	0,103	0,105
ID_12	rep	GRP_3	NO	1	SUB	F	1	2,290	2,234	2,347	309,286	531,000	0,103	0,100	0,103
ID_13	rep	GRP_4	YES	DOM	DOM	M	4	2,740	2,226	3,254	174,250	703,000	0,106	0,104	0,107
ID_14	rep	GRP_3	YES	DOM	DOM	M	3	1,207	1,248	1,166	146,647	713,333	0,102	0,100	0,110
ID_15	rep	GRP_4	YES	ELD	SUB	M	3	5,637	4,915	6,359	207,420	709,750	0,104	0,103	0,105
ID_16	rep	GRP_3	NO	ELD	SUB	M	2	4,272	3,128	5,416	268,150	643,333	0,103	0,102	0,104
ID_17	rep	GRP_4	YES	2	SUB	M	2	1,241	1,000	1,483	51,448	678,000	0,103	0,102	0,110
ID_18	rep	GRP_3	NO	2	SUB	M	2	1,200	0,750	1,650	261,212	618,000	0,107	0,102	0,110

ID_19	rep	GRP_3	NO	1	SUB	M	1	3,482	1,703	5,261	160,211	538,500	0,103	0,100	0,106
ID_20	rep	GRP_4	YES	DOM	DOM	F	4	1,846	0,604	3,088	330,977	790,500	0,103	0,102	0,103
ID_21	rep	GRP_4	YES	ELD	SUB	F	3	1,525	0,519	2,532	267,986	681,000	0,101	0,100	0,101
ID_22	rep	GRP_4	NO	2	SUB	F	2	4,386	0,827	7,946	74,118	636,500	0,103	0,101	0,110
ID_23	rep	GRP_4	NO	1	SUB	F	1	2,993	2,483	3,503	219,600	519,800	0,100	NA	NA
ID_24	rep	GRP_4	NO	1	SUB	M	1	5,759	4,170	7,349	217,559	560,250	0,104	0,100	0,110
ID_25	rep	GRP_6	YES	DOM	DOM	F	8	4,018	3,133	4,903	199,744	763,750	0,106	0,102	0,109
ID_26	rep	GRP_5	NO	ELD	SUB	F	3	2,596	0,404	4,789	396,699	724,167	0,103	NA	NA
ID_27	rep	GRP_5	NO	2	SUB	F	2	0,856	0,628	1,084	148,712	591,333	0,104	0,101	0,105
ID_28	rep	GRP_5	NO	1	SUB	F	1	1,827	1,746	1,907	450,082	725,625	0,105	0,100	0,110
ID_29	rep	GRP_5	NO	DOM	DOM	M	3	0,436	0,109	0,763	195,577	822,125	0,104	0,100	0,107
ID_30	rep	GRP_8	YES	ELD	SUB	M	3	2,989	2,443	3,536	181,731	836,250	0,104	0,101	0,107
ID_31	rep	GRP_8	YES	DOM	DOM	M	3	0,268	0,106	0,431	188,835	806,250	0,107	0,101	0,110
ID_32	rep	GRP_8	YES	2	SUB	M	2	2,072	0,499	3,644	209,455	704,000	0,110	NA	NA
ID_33	rep	GRP_5	NO	ELD	SUB	M	2	2,311	1,958	2,664	136,388	636,600	0,107	0,105	0,109
ID_34	rep	GRP_5	NO	1	SUB	M	1	3,893	3,062	4,724	436,840	707,750	0,105	0,104	0,106
ID_36	rep	GRP_6	NO	2	SUB	F	2	6,482	5,829	7,136	131,830	689,000	0,108	0,106	0,109

ID_37	rep	GRP_6	NO	1	SUB	F	1	4,519	3,940	5,097	331,600	543,000	0,107	0,104	0,110
ID_38	rep	GRP_6	NO	ELD	SUB	M	3	2,314	0,871	3,758	320,094	733,500	0,106	0,106	0,106
ID_39	rep	GRP_6	NO	2	SUB	M	2	2,114	1,239	2,989	139,376	562,400	0,107	0,105	0,109
ID_40	rep	GRP_6	NO	1	SUB	M	1	4,870	3,864	5,876	50,680	440,000	0,108	0,103	0,109
ID_41	rep	GRP_5	NO	DOM	DOM	F	7	2,536	1,815	3,257	56,325	825,111	0,109	0,107	0,110
ID_42	rep	GRP_7	YES	DOM	DOM	F	7	3,619	1,302	5,936	584,662	670,667	0,110	0,109	0,110
ID_43	rep	GRP_7	NO	ELD	SUB	F	4	1,498	0,754	2,242	57,030	718,000	0,101	NA	NA
ID_44	rep	GRP_7	NO	2	SUB	F	2	1,565	1,216	1,915	135,882	599,800	0,102	NA	NA
ID_45	rep	GRP_7	NO	1	SUB	F	1	4,745	3,515	5,975	54,409	560,286	0,107	0,104	0,110
ID_46	rep	GRP_7	NO	ELD	SUB	M	3	1,026	0,725	1,327	161,143	767,250	0,105	NA	NA
ID_47	rep	GRP_7	NO	2	SUB	M	2	0,812	0,567	1,056	NA	615,333	no	no	no
ID_48	rep	GRP_7	NO	1	SUB	M	1	3,866	3,372	4,360	338,727	539,600	0,106	0,102	0,110
ID_49	rep	GRP_2	NO	ELD	SUB	F	3	3,855	3,731	3,979	42,353	765,000	0,102	NA	NA
ID_50	rep	GRP_2	NO	2	SUB	F	2	1,971	0,621	3,322	128,341	759,500	0,107	0,101	0,109
ID_51	rep	GRP_2	NO	ELD	SUB	M	3	0,462	0,219	0,705	296,468	784,800	0,105	0,104	0,106
ID_52	rep	GRP_2	NO	2	SUB	M	2	3,634	3,216	4,052	261,032	774,667	0,105	0,101	0,109
ID_53	rep	GRP_2	NO	1	SUB	M	1	5,589	4,497	6,680	135,992	713,667	0,103	0,101	0,106
ID_54	rep	GRP_8	NO	ELD	SUB	F	2	9,539	8,526	10,552	95,078	542,667	0,104	0,102	0,106
ID_55	rep	GRP_8	YES	DOM	DOM	F	1	11,238	11,200	11,276	244,665	581,500	0,106	0,101	0,110

ID_56	rep	GRP_8	NO	1	SUB	M	1	7,343	6,923	7,762	71,667	578,500	0,108	0,102	0,109
ID_57	rep	GRP_9	NO	ELD	SUB	F	3	1,707	0,609	2,806	NA	730,833	no	no	no
ID_58	rep	GRP_9	NO	2	SUB	F	2	1,834	0,763	2,905	108,991	637,250	0,109	NA	NA
ID_59	rep	GRP_9	NO	1	SUB	F	1	1,150	0,925	1,376	39,057	579,500	0,106	0,100	0,107
ID_60	rep	GRP_9	YES	DOM	DOM	M	NA	0,760	0,412	1,107	NA	724,667	no	no	no
ID_61	rep	GRP_9	NO	ELD	SUB	M	4	0,978	0,879	1,078	83,883	731,000	0,103	NA	NA
ID_62	rep	GRP_9	NO	2	SUB	M	2	0,158	0,109	0,206	156,000	695,000	0,105	NA	NA
ID_63	rep	GRP_9	NO	1	SUB	M	1	0,805	0,324	1,286	43,307	568,200	0,105	0,103	0,109
ID_64	rep	GRP_1	YES	DOM	DOM	F	7	2,460	2,355	2,565	256,909	741,500	0,110	NA	NA
ID_65	rep	GRP_9	YES	DOM	DOM	F	7	5,960	3,216	8,703	NA	686,167	no	no	no
ID_66	rep	GRP_6	YES	DOM	DOM	M	7	2,302	1,886	2,718	225,421	741,250	0,107	NA	NA
ID_67	rep	GRP_7	YES	DOM	DOM	M	5	0,809	0,410	1,209	204,435	672,167	0,108	0,104	0,110
ID_1	nonrep	GRP_1	NO	ELD	SUB	F	3	2,122	2,073	2,171	578,244	674,000	0,108	0,107	0,109
ID_2	nonrep	GRP_1	NO	2	SUB	F	2	0,813	0,091	1,536	83,628	611,000	0,107	0,104	0,110
ID_3	nonrep	GRP_1	NO	1	SUB	F	1	2,209	0,705	3,712	NA	550,000	no	no	no
ID_4	nonrep	GRP_1	YES	DOM	DOM	M	NA	4,386	3,051	5,720	602,913	NA	0,103	NA	NA
ID_5	nonrep	GRP_1	NO	ELD	SUB	M	4	1,961	1,224	2,698	NA	696,000	no	no	no
ID_6	nonrep	GRP_1	NO	2	SUB	M	2	1,308	0,337	2,278	NA	645,000	no	no	no
ID_7	nonrep	GRP_1	NO	1	SUB	M	1	4,938	2,431	7,445	71,321	548,000	0,106	NA	NA
ID_8	nonrep	GRP_2	YES	DOM	DOM	M	NA	1,745	1,202	2,288	NA	817,000	no	no	no
ID_9	nonrep	GRP_2	YES	DOM	DOM	F	7	1,037	0,616	1,458	NA	781,000	no	no	no
ID_10	nonrep	GRP_3	NO	DOM	DOM	F	5	1,556	0,367	2,745	113,727	655,000	0,110	0,110	0,110
ID_11	nonrep	GRP_3	NO	ELD	SUB	F	2	1,810	1,060	2,560	NA	634,000	no	no	no
ID_12	nonrep	GRP_3	NO	1	SUB	F	1	1,940	1,852	2,028	193,211	574,000	0,107	0,101	0,109
ID_13	nonrep	GRP_4	YES	DOM	DOM	M	4	3,978	0,916	7,040	231,193	733,000	0,109	NA	NA
ID_14	nonrep	GRP_3	YES	DOM	DOM	M	3	1,853	1,207	2,500	168,545	731,000	0,107	0,101	0,110

ID_15	nonrep	GRP_4	YES	ELD	SUB	M	3	1,145	0,698	1,593	84,808	736,000	0,105	0,104	0,107
ID_16	nonrep	GRP_3	NO	ELD	SUB	M	2	1,345	0,364	2,327	47,647	688,000	0,102	0,101	0,104
ID_18	nonrep	GRP_3	NO	2	SUB	M	2	3,327	3,023	3,631	53,398	666,000	0,108	0,106	0,110
ID_19	nonrep	GRP_3	NO	1	SUB	M	1	2,301	2,013	2,588	25,234	567,000	0,107	NA	NA
ID_20	nonrep	GRP_4	YES	DOM	DOM	F	4	0,513	0,491	0,535	254,717	770,500	0,106	0,101	0,109
ID_21	nonrep	GRP_4	YES	ELD	SUB	F	3	0,918	0,878	0,957	139,010	666,000	0,101	NA	NA
ID_22	nonrep	GRP_4	NO	2	SUB	F	2	0,278	0,091	0,466	185,223	587,500	0,107	0,106	0,110
ID_23	nonrep	GRP_4	NO	1	SUB	F	1	0,905	0,118	1,693	360,921	475,000	0,106	0,105	0,107
ID_24	nonrep	GRP_4	NO	1	SUB	M	1	0,214	0,117	0,311	229,846	561,000	0,106	0,100	0,109
ID_25	nonrep	GRP_6	YES	DOM	DOM	F	8	0,719	0,464	0,973	NA	850,000	no	no	no
ID_26	nonrep	GRP_5	NO	ELD	SUB	F	3	2,129	0,947	3,310	637,620	743,000	0,106	0,102	0,110
ID_27	nonrep	GRP_5	NO	2	SUB	F	2	0,405	0,354	0,455	19,223	668,000	no	no	no
ID_28	nonrep	GRP_5	NO	1	SUB	F	1	2,641	1,818	3,464	512,083	777,500	0,104	0,100	0,108
ID_29	nonrep	GRP_5	NO	DOM	DOM	M	3	0,947	0,717	1,176	239,142	837,000	0,105	0,102	0,107
ID_30	nonrep	GRP_8	YES	ELD	SUB	M	3	1,350	1,315	1,385	83,119	780,000	0,107	0,100	0,109
ID_31	nonrep	GRP_8	YES	DOM	DOM	M	3	0,628	0,330	0,927	120,759	782,000	0,102	0,100	0,102
ID_32	nonrep	GRP_8	YES	2	SUB	M	2	0,831	0,645	1,017	32,264	721,500	0,106	NA	NA
ID_33	nonrep	GRP_5	NO	ELD	SUB	M	2	2,150	1,506	2,793	NA	723,500	0,102	0,100	0,104
ID_34	nonrep	GRP_5	NO	1	SUB	M	1	1,490	0,394	2,586	61,200	800,000	0,100	NA	NA

ID_35	nonrep	GRP_6	NO	ELD	SUB	F	3	0,964	0,662	1,266	NA	596,000	no	no	no
ID_36	nonrep	GRP_6	NO	2	SUB	F	2	1,259	0,984	1,535	225,000	755,000	0,104	NA	NA
ID_37	nonrep	GRP_6	NO	1	SUB	F	1	3,489	2,462	4,515	33,476	662,000	0,103	0,101	0,104
ID_38	nonrep	GRP_6	NO	ELD	SUB	M	3	0,341	0,334	0,349	298,899	829,500	0,109	NA	NA
ID_39	nonrep	GRP_6	NO	2	SUB	M	2	0,929	0,806	1,053	26,214	622,000	0,103	NA	NA
ID_40	nonrep	GRP_6	NO	1	SUB	M	1	3,700	3,490	3,910	33,333	504,500	0,108	0,100	0,108
ID_41	nonrep	GRP_5	NO	DOM	DOM	F	7	0,091	0,091	0,091	NA	871,000	no	no	no
ID_42	nonrep	GRP_7	YES	DOM	DOM	F	7	1,987	0,217	3,757	NA	698,500	0,110	NA	NA
ID_43	nonrep	GRP_7	NO	ELD	SUB	F	4	0,155	0,108	0,202	196,200	753,000	0,100	NA	NA
ID_44	nonrep	GRP_7	NO	2	SUB	F	2	1,164	0,433	1,895	337,508	620,500	0,105	0,104	0,105
ID_45	nonrep	GRP_7	NO	1	SUB	F	1	2,755	2,353	3,158	NA	569,000	no	no	no
ID_46	nonrep	GRP_7	NO	ELD	SUB	M	3	1,526	0,944	2,107	502,018	786,000	0,109	NA	NA
ID_47	nonrep	GRP_7	NO	2	SUB	M	2	0,835	0,230	1,440	489,981	644,000	0,102	0,100	0,104
ID_48	nonrep	GRP_7	NO	1	SUB	M	1	2,095	0,863	3,326	396,000	560,000	0,105	0,100	0,110
ID_49	nonrep	GRP_2	NO	ELD	SUB	F	3	0,108	0,091	0,126	387,692	788,500	0,102	0,100	0,104
ID_50	nonrep	GRP_2	NO	2	SUB	F	2	0,549	0,462	0,635	32,079	806,000	0,101	0,101	0,104
ID_51	nonrep	GRP_2	NO	ELD	SUB	M	3	0,624	0,364	0,884	514,766	842,000	0,107	NA	NA
ID_52	nonrep	GRP_2	NO	2	SUB	M	2	2,616	2,080	3,152	245,143	798,000	0,105	0,105	0,105
ID_53	nonrep	GRP_2	NO	1	SUB	M	1	2,151	1,910	2,393	234,469	731,000	0,106	0,103	0,110
ID_54	nonrep	GRP_8	NO	DOM	DOM	F	2	0,951	0,433	1,469	96,923	546,000	0,104	0,103	0,110
ID_56	nonrep	GRP_8	NO	1	SUB	M	1	1,365	0,225	2,505	37,902	586,500	0,105	0,101	0,108
ID_58	nonrep	GRP_9	NO	2	SUB	F	2	4,097	3,810	4,385	NA	628,000	no	no	no

ID_59	nonrep	GRP_9	NO	1	SUB	F	1	3,301	2,373	4,230	171,346	563,000	0,104	0,104	0,108
ID_60	nonrep	GRP_9	YES	DOM	DOM	M	NA	0,712	0,708	0,716	NA	676,000	no	no	no
ID_61	nonrep	GRP_9	NO	ELD	SUB	M	4	3,804	3,315	4,294	NA	697,000	no	no	no
ID_62	nonrep	GRP_9	NO	2	SUB	M	2	1,982	1,343	2,620	28,235	678,000	0,102	NA	NA
ID_63	nonrep	GRP_9	NO	1	SUB	M	1	1,196	0,531	1,861	112,941	603,000	0,102	0,101	0,105
ID_64	nonrep	GRP_1	YES	DOM	DOM	F	7	1,203	0,838	1,568	614,019	738,000	0,107	NA	NA
ID_65	nonrep	GRP_9	YES	DOM	DOM	F	6	1,612	1,582	1,641	40,000	692,500	0,108	NA	NA
ID_66	nonrep	GRP_6	YES	DOM	DOM	M	7	1,378	0,835	1,920	NA	784,500	no	no	no
ID_67	nonrep	GRP_7	YES	DOM	DOM	M	5	0,492	0,136	0,847	388,771	691,000	0,106	0,100	0,110
